# Helicase LSH/Hells regulates kinetochore function, histone H3/Thr3 phosphorylation and centromere transcription during oocyte meiosis

**DOI:** 10.1038/s41467-020-18009-3

**Published:** 2020-09-08

**Authors:** Claudia Baumann, Wei Ma, Xiaotian Wang, Muthugapatti K. Kandasamy, Maria M. Viveiros, Rabindranath De La Fuente

**Affiliations:** 1 Department of Physiology and Pharmacology, College of Veterinary Medicine, University of Georgia, Athens, GA 30602 USA; 2 Regenerative Biosciences Center (RBC), University of Georgia, Athens, GA 30602 USA; 3 School of Basic Medical Sciences, Capital Medical University, 100069 Beijing, China; 4 Department of Genetics, University of Georgia, Athens, GA 30602 USA

**Keywords:** Centromeres, Kinetochores

## Abstract

Centromeres are epigenetically determined nuclear domains strictly required for chromosome segregation and genome stability. However, the mechanisms regulating centromere and kinetochore chromatin modifications are not known. Here, we demonstrate that LSH is enriched at meiotic kinetochores and its targeted deletion induces centromere instability and abnormal chromosome segregation. Superresolution chromatin analysis resolves LSH at the inner centromere and kinetochores during oocyte meiosis. LSH knockout pachytene oocytes exhibit reduced HDAC2 and DNMT-1. Notably, mutant oocytes show a striking increase in histone H3 phosphorylation at threonine 3 (H3T3ph) and accumulation of major satellite transcripts in both prophase-I and metaphase-I chromosomes. Moreover, knockout oocytes exhibit centromere fusions, ectopic kinetochore formation and abnormal exchange of chromatin fibers between paired bivalents and asynapsed chromosomes. Our results indicate that loss of LSH affects the levels and chromosomal localization of H3T3ph and provide evidence that, by maintaining transcriptionally repressive heterochromatin, LSH may be essential to prevent deleterious meiotic recombination events at repetitive centromeric sequences.

## Abstract (web summary)

Centromeres are the sites of kinetochore and inner centromere formation, which can be epigenetically regulated. Here, the authors reveal a role for the lymphocyte specific helicase LSH/Hells associated with pericentric heterochromatin formation in centromere stability and chromosome segregation at meiotic kinetochores.

## Introduction

Centromeric heterochromatin formation is essential for the control of gene expression, nuclear architecture, and chromosome segregation^1,2^. During mammalian meiosis, unique mechanisms are set in place in order to cope with the specific structural and functional requirements of chromosome segregation in the germ line^3,4^. Meiotic centromeres exhibit specialized functions that are essential to coordinate sister kinetochore cohesion and homologous chromosome bi-orientation at the metaphase-I (M-I) spindle with accurate chromosome segregation^5^. Germ cell-specific mechanisms of heterochromatin formation are, thus, critical to fulfill the segregation requirements of meiotic chromosomes^3,4^. Abnormal chromosome segregation during oocyte meiosis results in transmission of aneuploidy to the early embryo and is a significant cause of human trisomies^6^.

Centromeres are the sites of kinetochore and inner centromere formation, and in most species centromere structure and function are epigenetically regulated^2,7^. Histone H3 phosphorylation at threonine 3 (H3T3ph) is required to establish the inner centromere in both yeast and human cells^8,9^. Importantly, histone depletion in *Xenopus laevis* egg extracts indicates that the presence of nucleosomes is also essential for microtubule spindle formation^10^. The underlying mechanisms regulating distinct chromatin modifications at specific centromeric domains in model organisms such as budding yeast are beginning to be unraveled^11^. However, little is known about the molecular mechanisms regulating histone modifications required for meiotic centromere function.

The lymphocyte-specific helicase LSH/Hells is a DNA helicase essential for genome-wide DNA methylation as well as methylation of repetitive sequences in the mouse genome^12,13^. LSH contributes to the regulation of nucleosome density as well as the genomic distribution patterns of histone mono-methylation (H3K4me1) in mouse fibroblasts and neuronal precursors^14,15^. We previously demonstrated that LSH is essential for homologous chromosome synapsis and maintenance of chromosome stability in female^16^ and male^17^ germ cells. Our studies indicate that LSH is required for DNA methylation of major satellite sequences at pericentric heterochromatin in pachytene oocytes^16^. However, the molecular mechanisms of LSH function during meiosis remained to be established. Here, we define the function of LSH during mouse prophase-I and M-I, two key stages of oocyte meiosis essential to regulate accurate chromosome segregation. Our results indicate that LSH exhibits a dynamic chromosomal localization. At early meiotic prophase-I, it is an abundant nuclear protein required for the chromatin localization of histone deacetylase 2 (HDAC2) and DNMT-1. Notably, super-resolution chromatin analysis indicates that LSH is enriched at the inner centromere at late pachytene and at both the inner centromere and kinetochores of M-I and metaphase-II (M-II) stage oocytes. Targeted deletion induced a striking increase in the levels of H3T3ph in prophase-I and M-I oocytes. Using RNA fluorescence in situ hybridization (RNA-FISH), we provide the first evidence for the presence of major satellite transcripts at meiotic centromeric heterochromatin. Remarkably, *Lsh*^(−/−)^ oocytes exhibit a fourfold overexpression of centromeric transcripts. Our results indicate that LSH exhibits a previously unrecognized localization to the meiotic kinetochore and inner centromere and its targeted deletion affects the levels and chromosomal localization of H3T3ph, centromere transcription as well as abnormal chromosome segregation during oocyte meiosis. Importantly, our results provide evidence that through its prominent role in pericentric heterochromatin formation, LSH may be critical to prevent deleterious centromeric meiotic recombination. Our results have important implications to understand the long-sought mechanisms involved in the centromere effect during meiosis as well as the mechanisms of formation of human trisomies.

## Results

### LSH is required for meiotic kinetochore structure in pachytene oocytes

**Fig. 1:**
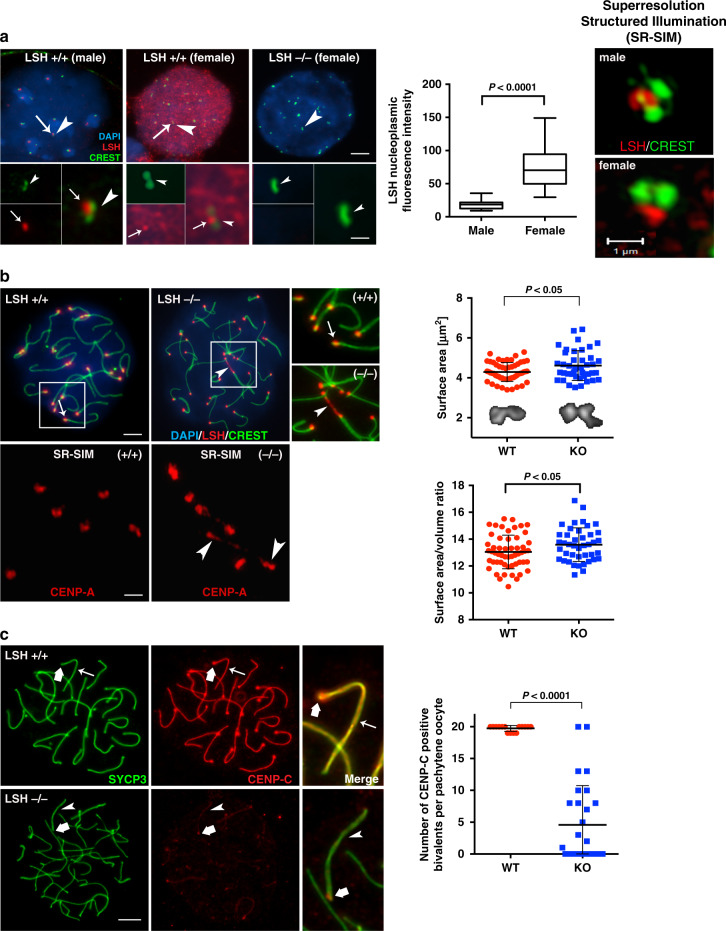
LSH is required for kinetochore structure and function during female meiotic prophase-I. **a** Sexual dimorphism in the patterns of LSH nuclear localization in mouse male and female germ cells. In pachytene spermatocytes, LSH (red) is present at the inner centromere (arrow) in close apposition with the kinetochores (arrowhead) detected by CREST (green; inset). In pachytene oocytes, LSH (red) exhibits a diffuse nucleoplasmic localization, resulting in a significant increase in fluorescence intensity, but is also enriched at the inner centromere (arrow; inset). Note the absence of LSH staining in Lsh^−/−^ pachytene oocytes. Top scale bar = 5 μm. Inset scale bar = 1 μm. Box plots depict the median value as well as upper and lower quartiles with whiskers representing the minimum and maximum values observed in *n* = 15 male and *n* = 15 female pachytene cells examined over three independent experiments. Unpaired *t*-test, two tailed and resulted in *P* < 0.0001. Super-resolution structured illumination (SR-SIM) confirms the localization of LSH to the inner centromere in both male and female germ cells at the pachytene stage (scale bar = 1 μm). **b** Loss of LSH function induces abnormal elongation of chromatin fibers stained with CENP-A in pachytene oocytes (arrowheads) and decondensed kinetochores with increased surface area. Data are presented as the mean ± SD of wild-type (*n* = 55) and LSH^−/−^ (*n* = 41) paired kinetochores over three independent biological replicates. Unpaired *t*-test, two tailed and resulted in *P* = 0.011 (Surface area) and *P* = 0.0373 (surface area/volume ratio). Scale bars = 2 μm. **c** CENP-C (red) is initially present at the meiotic kinetochores (bold arrow) but extends throughout the synaptonemal complex (green) of synapsed chromosomes (thin arrow) in wild-type pachytene oocytes (*n* = 15). CENP-C is detectable at the kinetochore in LSH^−/−^ oocytes (*n* = 28) but is absent from partially synapsed or loosely paired bivalents in mutant oocytes (arrowhead). Data are expressed as the mean ± SD over three independent replicates. Unpaired *t*-test, two tailed and resulted in *P* < 0.0001. Scale bar = 2 μm.

We previously demonstrated that LSH is transiently enriched at centromeric heterochromatin in zygotene-stage oocytes^16^. Notably, comparison of male and female germ cells revealed a sexual dimorphism in the patterns of LSH nuclear localization. In pachytene oocytes, LSH (red) is an abundant nuclear protein that becomes enriched at the inner centromere where it is partially co-localized with the CREST signals (green) detected at sister kinetochores (Fig. 1a). By contrast, LSH is significantly reduced in the nucleoplasm of pachytene spermatocytes but is detectable at the inner centromere domain at the late pachytene stage (Fig. 1a). Analysis of chromosome structural proteins, following detergent (Triton X) extraction and fast hypotonic chromatin decondensation under conditions that preserve kinetochore structure^18^ combined with super-resolution structured illumination (SR-SIM)^19^, resolves the shape of bi-lobed, fused kinetochores at the pachytene stage with prominent LSH localization at the inner centromere in both male and female germ cells (Fig. 1a). Thus, LSH is present at the inner centromere at the pachytene stage in male and female germ cells, but it also exhibits a dynamic and sexually dimorphic chromosomal localization during mammalian meiosis.

To determine whether LSH plays a role in the regulation of kinetochore structure and function during meiotic prophase-I, we compared the expression and chromosomal localization of key component molecules of the kinetochore complex in wild-type and Lsh^*−/−*^ mutant oocytes^16^. As expected, centromeric protein-A (CENP-A) is localized at the kinetochores of fully synapsed bivalents in wild-type pachytene oocytes (Fig. 1b), and SR-SIM resolved a single CENP-A focus on each strand of the lateral elements of the synaptonemal complex (SC) (Supplementary Fig. 1A). CENP-A is present in mutant oocytes. However, loss of LSH function results in the formation of stretched chromatin fibers stained with CENP-A and abnormal interactions between decondensed kinetochores (Fig. 1b; arrowheads). Quantitative 3D super-resolution analysis revealed a significant increase in both kinetochore surface area and surface area/volume ratio in mutant oocytes (Fig. 1b and Supplementary Movies 1 and 2) consistent with a role for LSH in meiotic kinetochore structure. CENP-C is a major structural component of mammalian kinetochores^2,7^. Moreover, kinetochore elongation has been shown to increase CENP-C binding to chromosomes^20^. Therefore, we determined the patterns of CENP-C localization in *Lsh*^*−/−*^ mutant oocytes. In wild-type oocytes, CENP-C is present as a prominent kinetochore protein at all stages of meiotic prophase-I (Supplementary Fig. 1B). Notably, at the pachytene stage, CENP-C undergoes a striking, albeit transient, redistribution toward the SC (Fig. 1c) where it may be recruited by the presence of minor satellite DNA sequences that can be detected at the central element of the SC in pachytene oocytes (Supplementary Fig. 1B, C). CENP-C is detectable at the kinetochore of mutant oocytes. However, only negligible CENP-C staining can be detected at the SC of partially synapsed bivalents in mutant oocytes (Fig. 1c) indicating that abnormal synapsis in mutant oocytes prevents the association of CENP-C with the SC. These results indicate that LSH is critical for meiotic kinetochore structure and function during female meiotic prophase-I. Importantly, we provide evidence that CENP-C exhibits a striking redistribution during homologous chromosome synapsis, suggesting a potential meiosis-specific role in mouse oocytes.

**Fig. 2:**
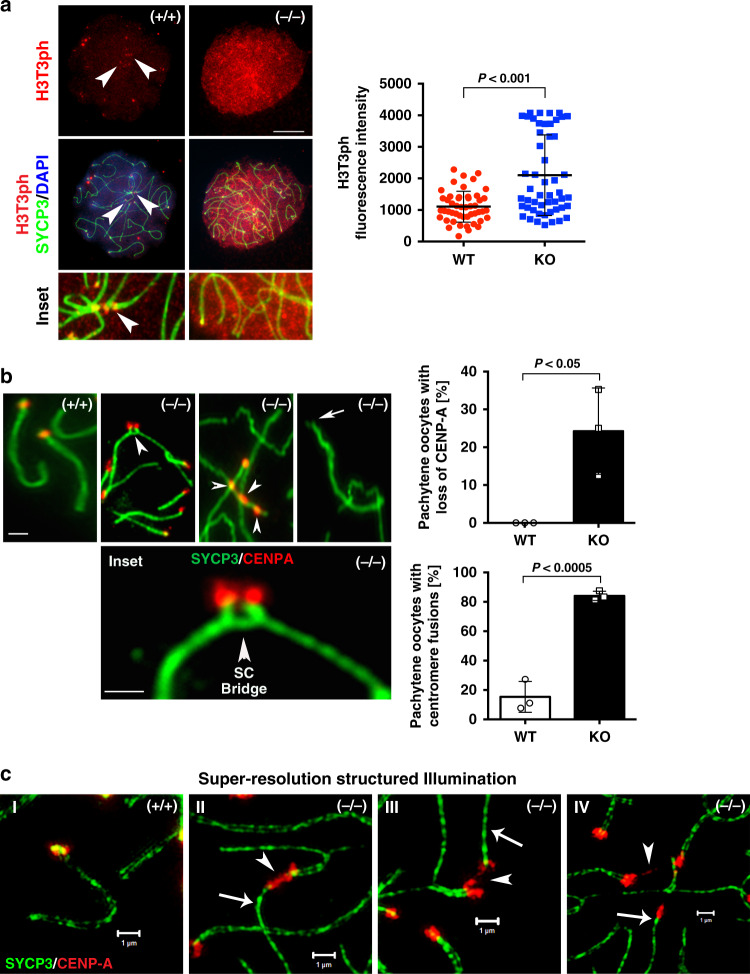
LSH^(−/−)^ oocytes exhibit increased H3T3 phosphorylation and kinetochore fusions between paired bivalents and asynapsed chromosomes. **a** Histone H3 phosphorylation at threonine 3 (H3T3ph; red) exhibits a kinetochore localization (arrowheads) in fully synapsed bivalents of wild-type pachytene oocytes (*n* = 49). By contrast, LSH^−/−^ oocytes (*n* = 51) exhibit a significant increase in the levels of H3T3ph throughout the nucleoplasm and abnormal chromosome synapsis. Oocytes were exposed to TX-100 detergent extraction before immunochemistry. Data represent the mean ± SD over three independent replicates. Mann–Whitney test, two tailed and resulted in *P* < 0.0001. Scale bar = 10 μm. **b** CENP-A staining (red) reveals the presence of fused sister kinetochores at properly synapsed bivalents in wild-type oocytes (scale bar = 2 μm). Notably, LSH^−/^^−^ oocytes exhibit abnormal exchange of chromatin fibers between paired bivalents and asynapsed chromosomes resulting in the formation of a synaptonemal complex (SC) bridge (arrowhead), resembling a proximal exchange near the kinetochores of mutant oocyte bivalents (inset; scale bar = 2 μm). LSH^−/−^ oocytes also exhibit asynapsed chromosomes with multiple CENP-A signals (arrowheads) as well as chromosomes completely lacking CENP-A staining (arrow) suggesting the presence of large scale, chromosomal exchanges proximal to the centromere. Data are expressed as the mean ± SD of three independent experimental replicates with (*n* = 59) wild-type and (*n* = 36) KO oocytes per replicate. Unpaired *t*-tests, two tailed and resulted in *P* = 0.0004 (fusions) and *P* = 0.0212 (loss of CENP-A). **c** Super-resolution structured illumination revealed the presence of kinetochore fusions in mutant oocytes. I Fully synapsed bivalent in wild-type pachytene oocytes. II Abnormal fusions (arrowhead) between a partially synapsed bivalent and an adjacent asynapsed chromosome (arrow) in mutant oocytes. III Fused kinetochores begin to stretch as asynapsed chromosomes (arrow) pull apart. IV Presence of asynapsed chromosomes (arrow), in which the CENP-A signal establishes contact with the centromere of a partially synapsed bivalent induce stretched chromatin fibers (arrowhead; right), suggesting abnormal centromere fusions and chromatin stretching as a mechanism for the loss of CENP-A signals in mutant pachytene oocytes (scale bar = 1 μm).

H3T3ph is a prominent posttranslational modification required for inner centromere specification^8,9^. However, the mechanisms regulating inner centromere chromatin modifications are not clear at present. In wild-type oocytes, H3T3ph is localized at the kinetochore in pachytene oocytes with fully synapsed bivalents (Fig. 2a; arrowheads and inset). Notably, LSH mutant oocytes exhibit a significant increase in the levels of H3T3ph where this histone mark becomes a diffuse nucleoplasmic signal (Fig. 2a). High-resolution chromatin analysis indicates the presence of abnormal chromosome contacts and interactions mediated by protruding chromatin fibers, proximal to the kinetochore (Fig. 2b). These fibers extend from an asynapsed chromosome and establish contact with a synapsed bivalent forming an SC bridge resembling a proximal exchange (Fig. 2b and inset). Notably, mutant pachytene oocytes also contained asynapsed chromosomes bearing up to three CENP-A signals (arrowheads) as well as bivalents completely lacking CENP-A (arrow) indicating a significant loss of kinetochore domains in 24.3% of pachytene oocytes (Fig. 2b). Detection of centromere fusions in >80% of pachytene oocytes and loss of CENP-A signal suggest the presence of major abnormal chromosomal exchanges in mutant oocytes. SR-SIM revealed the formation of different types of centromere defects in mutant oocytes (Fig. 2c). The most prominent defects observed were centromere fusions (arrowheads) between partially synapsed bivalents and adjacent asynapsed chromosomes (Fig. 2c, panel II thin arrow) and confirmed a high frequency of asynapsed chromosomes that exhibited abnormal contacts with the centromere domains of synapsed bivalents resulting in the formation of stretched chromatin fibers stained with CENP-A (Fig. 2c, panels III, IV). This suggests that centromere fusions and stretching of chromatin fibers may result in the subsequent loss of CENP-A signals in a few chromosomes. By contrast, wild-type oocytes exhibited a single CENP-A signal on each strand of the lateral elements of the SC (Fig. 2c, panel I). These results indicate that the loss of LSH function induces a striking increase in the levels of H3T3 phosphorylation and results in the formation of large-scale chromosomal exchanges proximal to the centromere, consistent with abnormal centromeric recombination events during meiotic prophase-I.

### Loss of centromere cytosine methylation in Lsh^(−/−)^ pachytene oocytes

**Fig. 3:**
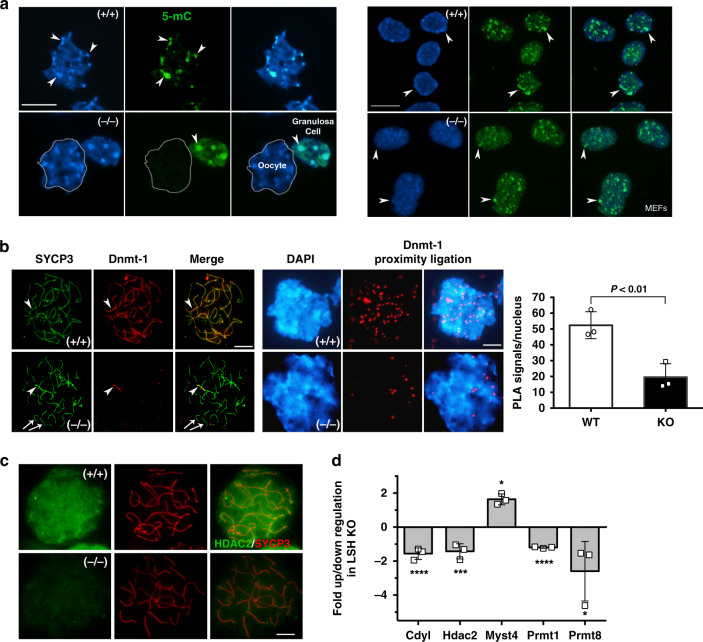
LSH knockout results in reduced DNMT-1 and HDAC2 in pachytene oocytes. **a** LSH exhibits a germ cell-specific role in centromere cytosine methylation. 5-methyl Cytosine (5-mC; green) is enriched at pericentric heterochromatin domains (arrowheads) detected by bright DAPI staining in wild-type pachytene oocytes. LSH mutant oocytes lack 5-mC staining at pericentric heterochromatin. However, granulosa cells of LSH mutant ovaries exhibit normal levels of 5-mC staining at heterochromatin domains. No differences were detected in the patterns of 5-mC staining in somatic cells (embryonic fibroblasts; MEFs) of wild-type and LSH mutant embryos on day E14 (scale bar = 10 μm). **b** DNMT-1 associates with the synaptonemal complex in wild-type late pachytene oocytes (*n* = 53) on embryonic day E17.5. However, confocal microscopy and proximity ligation assays revealed a significant reduction in DNMT-1 in LSH^−/−^ oocytes (*n* = 52). Data show the mean ± SD of three independent replicates (scale bar = 5 μm). Unpaired *t*-test, two tailed and resulted in *P* = 0.0093. **c** HDAC2 is an abundant nucleoplasmic protein in wild-type oocytes at the pachytene stage. LSH knockout induces a significant reduction of HDAC2 in pachytene oocytes. **d** LSH mutant ovaries also exhibit a significant reduction in transcripts for HDAC2 (***P = 0.0007), the chromodomain CDYL (****P = 0.0002) and the arginine methyltransferases PRMT-1 (****P < 0.0001) and PRMT-8 (*P = 0.0237), and show increased transcripts for the histone acetyl transferase MYST4 (*P = 0.0266). Plot of expression values represent the log2 fold change showing significant differences in transcript levels after analysis of 84 chromatin-modifying enzymes compared to housekeeping genes (Actin, Gapdh, Hsp90ab1, Gusb) as internal controls. Data represent the mean ± SD of three independent replicates (scale bar = 5 μm). Statistical analysis was conducted using unpaired *t*-tests, two tailed.

Our previous studies indicate that targeted deletion of LSH results in loss of DNA methylation at major satellite sequences during meiosis^16^. However, the mechanisms involved in this process remained to be established. Therefore, we interrogated the transcriptional profile of LSH mutant oocytes using array profiling for epigenetic modifying enzymes as well as single-cell proximity ligation assays for the analysis of endogenous protein complexes^21^. In wild-type oocytes, 5-methyl Cytosine (5-mC) is a prominent marker of pericentric heterochromatin (Fig. 3a; arrowheads). Notably, LSH targeted deletion results in lack of 5-mC nuclear localization in mutant oocytes but not in the somatic granulosa cells of mutant ovaries (Fig. 3a). In addition, no differences were detected in the patterns of 5-mC staining in embryonic fibroblasts from wild-type and mutant fetuses indicating the existence of a germ cell-specific role for LSH in the establishment of 5-mC at pericentric heterochromatin during female meiosis.

To determine the mechanisms by which LSH, a helicase protein with no methyl transferase activity^14^, regulates DNA methylation, we used single-cell proximity ligation to quantify the levels of DNMT-1, a key component of the DNA methylation machinery. Our protocol for fast chromatin decondensation revealed that DNMT-1 (red) is co-localized with the SC protein SYCP3 in wild-type oocytes (Fig. 3b). By contrast, no DNMT-1 was detected in the majority of mutant oocytes except for some residual staining in one or two partially synapsed bivalents. Consistent with these results, proximity ligation assays (PLA) revealed a threefold reduction in the nuclear localization of DNMT-1 in mutant oocytes (Fig. 3b). The rolling-circle amplification of the PLA generates a signal only when a single DNMT-1 molecule is specifically detected by the antibody and thus appears as nuclear foci allowing for quantification of endogenous protein^21^. Importantly, transcriptional profiling using a panel of 84 key chromatin-modifying enzymes and confocal microscopy revealed that loss of LSH induced a significant reduction in both transcript and protein signal for HDAC2 as well as reduced transcripts for CDYL a chromodomain Y-like transcriptional repressor and the arginine methyltransferases Prmt-1 and Prmt-8 (Fig. 3c, d and Supplementary Fig. 2A, B). Mutant oocytes also exhibited a significant increase in transcripts for the histone acetyl transferase MYST4. These results indicate that LSH mutant oocytes exhibit a reduction in both DNMT-1 and HDAC2 at meiotic prophase-I in addition to significant changes in the expression of key chromatin-modifying enzymes that may result in global histone hyperacetylation and increased histone crotonylation, a newly identified histone modification associated with active transcription^22^.

### LSH is required for accurate meiotic chromosome segregation

**Fig. 4:**
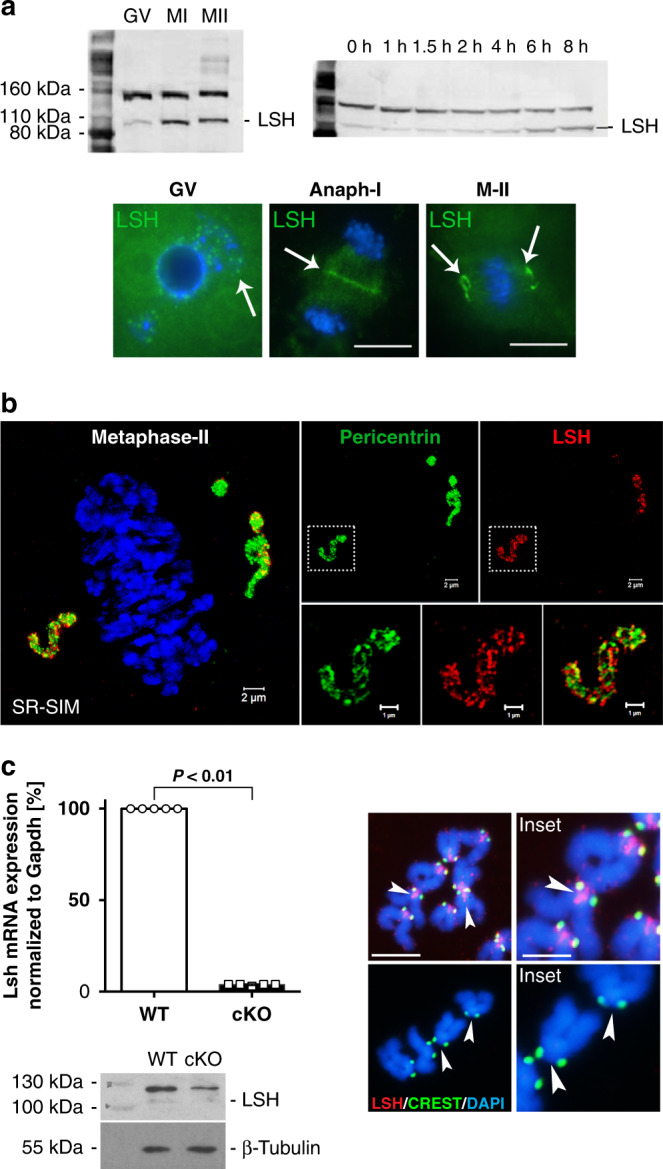
LSH is present at the meiotic kinetochore and spindle poles during oocyte maturation. **a** LSH exhibits a dynamic subcellular localization during oocyte meiotic maturation. Western blot detects LSH as a 110 KDa protein in mouse oocytes at the germinal vesicle (GV) stage (100 oocytes/lane). LSH is subject to translational regulation at metaphase-I (M-I) resulting in increased expression levels 6–8 h after the onset of GV breakdown. LSH is localized to nuclear speckles (arrow) in GV stage oocytes, but exhibits a dynamic redistribution to the mid-body during anaphase-I and to the meiotic spindle poles in metaphase-II (M-II) stage oocytes. **b** Super-resolution structured illumination (SR-SIM) resolves the co-localization of LSH (red) with pericentrin (green) at spindle poles in M-II stage oocytes (scale bar = 2 μm). See insets (scale bar = 1 μm). **c** ZP3-*Cre* oocyte conditional deletion of LSH. Real-time PCR revealed a significant reduction in LSH transcripts in fully grown conditionally deleted cKO oocytes. Data are expressed as the mean ± SD of five experimental replicates with (*n* = 50) wild-type and (*n* = 50) cKO oocytes per replicate. Statistical analysis was performed using unpaired *t*-tests, two tailed and resulted in *P* < 0.0001. Loss of LSH protein was confirmed by western blot. Analysis of chromosomal spreads revealed that LSH is present at the kinetochore and inner centromere domains in metaphase-II stage chromosomes from wild-type oocytes, but is absent from chromosomes of cKO oocytes (scale bar = 5 μm). Inset scale bar (2 μm).

Conventional LSH knockout mice die shortly after birth^23^ precluding its functional analysis in mature oocytes. Therefore, we generated an oocyte conditional deletion to determine its function in fully grown oocytes. In wild-type oocytes at the germinal vesicle (GV) stage, LSH is detected as a 110-KDa protein (Fig. 4a and Supplementary Fig. 3A) that exhibits a striking increase in expression levels at the M-I stage of meiosis ~6–8 h after germinal vesicle breakdown (GVBD) with similar levels maintained in mature oocytes at the M-II stage. At the GV stage, LSH exhibits both nuclear and cytoplasmic localization. Notably, this helicase protein undergoes a major transition in subcellular localization from nuclear speckles in oocytes that exhibit a surrounded nucleolus configuration, to the meiotic spindle mid-body at anaphase-I, and subsequently to the spindle poles at the M-II stage (Fig. 4a; lower panel). SR-SIM indicates that LSH (red) co-localizes with pericentrin (green), a bona fide marker of spindle poles in M-II oocytes (Fig. 4b; insets and Supplementary Movie 3). Importantly, analysis of chromosome spreads revealed that in addition to the spindle poles, LSH is enriched at the meiotic kinetochore and inner centromere of M-II stage oocytes (Fig. 4c; arrowheads).

**Fig. 5:**
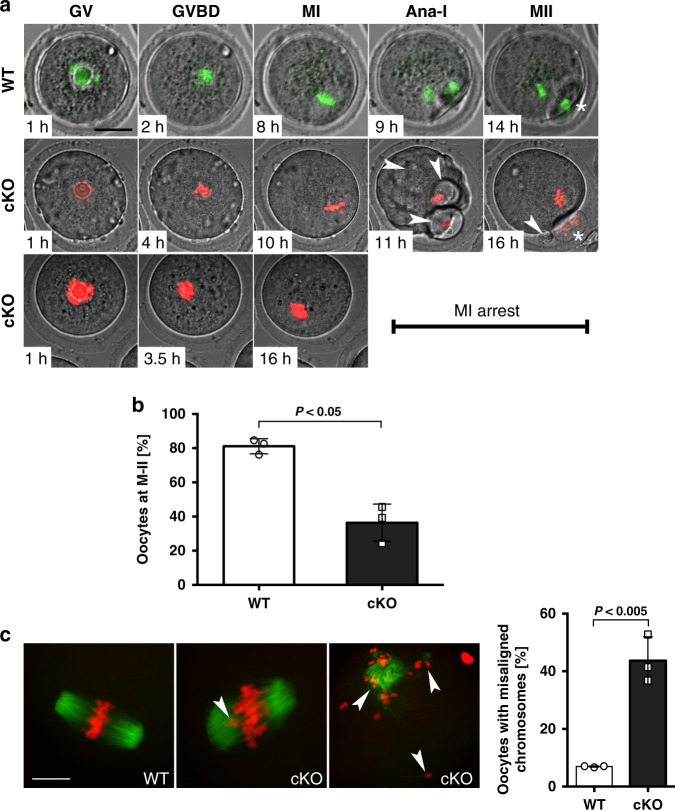
Conditional deletion of LSH disrupts meiotic progression and chromosome segregation in preovulatory oocytes. **a** Live-cell imaging revealed a delayed onset of germinal vesicle breakdown (GVBD) in LSH-cKO oocytes and defects in oocyte maturation including membrane ruffling (arrowheads), abnormal cytokinesis, and splitting of the first polar body (arrowhead/asterisk) in a subpopulation of oocytes that reached the metaphase-II stage, while some oocytes remained at metaphase-I for up to 16 h of in vitro maturation. Chromosomes in wild-type oocytes were labeled with recombinant histone H2B-GFP (green), cKO oocytes were microinjected with H2B-RFP (red). Scale bar (40 μm). **b** Proportion of oocytes reaching the metaphase-II stage in wild-type (*n* = 218) and cKO (*n* = 198) oocytes. LSH knockout induced a significant reduction in oocyte maturation to the metaphase-II stage. Data are shown as the mean ± SD of three independent replicates. Statistical analysis was performed using unpaired *t*-tests, two tailed and resulted in *P* = 0.0027. **c** Mutant oocytes exhibit chromosome congression defects at metaphase-I (arrowhead) and meiotic spindle instability at the metaphase-II stage (arrowheads) resulting in a significant increase in the proportion of in vitro matured oocytes with misaligned chromosomes. Chromosomes were stained with DAPI and pseudo colored in red and microtubules were stained for acetylated tubulin (green). Data are presented as the mean ± SD of three replicates with wild-type (*n* = 86) and cKO (*n* = 84) oocytes. Scale bar (15 μm). Statistical analysis was performed using unpaired *t*-tests, two tailed and resulted in *P* = 0.0015.

To induce the functional ablation of LSH in preovulatory oocytes, we crossed homozygous female mice (C57BL/6NTac-Hells^tm1a (EUCOMM)wtsi^) carrying floxed *Lsh* alleles with ZP3-Cre;*Lsh*^*flox/−*^ males to generate ZP3-Cre;*Lsh*^*−/−*^ conditional KO (cKO) mice. Deletion of exon 11 disrupts the C-terminal helicase domain of LSH (Supplementary Fig. 3B). Real-time PCR revealed a significant (*P* < 0.001, unpaired *t*-test; 96.2%) reduction in *Lsh* transcripts compared to controls and protein ablation was confirmed by western blot analysis (Fig. 4c). Wild-type oocytes revealed LSH staining at the inner centromere and kinetochore domains of M-II stage oocytes. Importantly, no LSH protein was detected in the chromosomes of cKO oocytes indicating efficient deletion (Fig. 4c and Supplementary Fig 3C). LSH knockout in preovulatory oocytes had a striking effect on meiotic progression. Live-cell imaging revealed a 2-h delay in meiotic resumption (onset of GVBD) in cKO oocytes compared to controls (Fig. 5a and Supplementary Fig 4A) and a significant reduction in the proportion of M-II stage oocytes after in vitro maturation. Less than 40% of mutant oocytes progressed into M-II, albeit with delayed kinetics, showed membrane ruffling at anaphase-I (arrowheads) and a split polar body at M-II (arrowhead), suggesting abnormal cytokinesis. Notably, 62% of cKO oocytes exhibited M-I arrest (Fig. 5b and Supplementary Movies 4 and 5) and >40% showed misaligned chromosomes (Fig. 5c, middle panel), while some M-II oocytes failed to maintain a bipolar meiotic spindle after completion of meiotic maturation (Fig. 5c, right panel). LSH knockout also results in a significant reduction (*P* < 0.01; unpaired *t*-test, two tailed) in the average litter size of transgenic females and subfertility after natural mating (Supplementary Fig. 3C). These results indicate that LSH plays a critical role in meiotic progression and provide the first evidence for a potential link between centromeric chromatin remodeling, kinetochore function, and meiotic spindle stability.

### LSH regulates H3/Thr3ph and meiotic centromere transcription

**Fig. 6:**
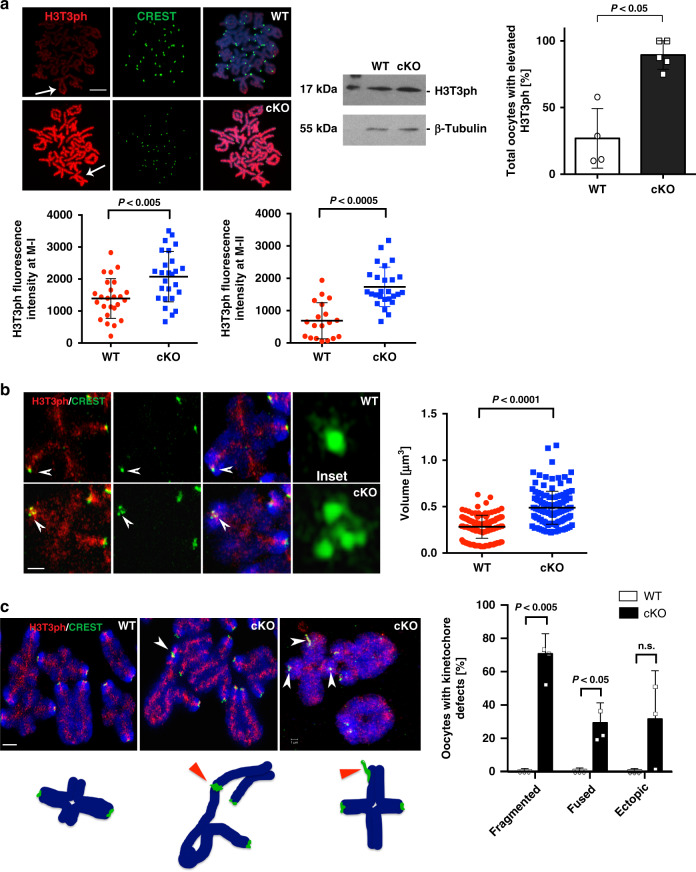
LSH knockout in preovulatory oocytes induces increased H3T3 phosphorylation and abnormal kinetochore structure. **a** Wild-type (WT) oocyte showing H3T3ph localization (red) to the inter-chromatid axis of metaphase-I chromosome bivalents (arrow), fused sister kinetochores are stained with CREST (green). DNA was counterstained with DAPI (blue) scale bar = 5 μm. Oocytes from *Lsh*-cKO mutant females exhibit a striking increase in the levels of H3T3ph (arrow) decorating the entire chromosome. Higher levels of H3T3ph were confirmed by western blot analysis and result from a significant increase in the proportion of oocytes with elevated H3T3ph at metaphase-I and metaphase-II stage (bar plot; *P* = 0.0063). Fluorescence intensity at M-I and M-II (scatter plots; *P* = 0.0012 and *P* < 0.0001). Data shown as the mean ± SD with wild-type (*n* = 44) and cKO (*n* = 52) oocytes in four independent experimental replicates. Statistical analysis was performed using unpaired *t*-tests, two tailed. **b** Super-resolution structured illumination (SR-SIM) reveals the compact structure of fused sister kinetochores in metaphase-I bivalents of wild-type oocytes (inset). By contrast, sister kinetochores fail to fuse, are decondensed, and frequently fragment in *Lsh*-cKO oocytes (inset). 3D SR-SIM analysis indicates that abnormal kinetochore structure in mutant oocytes results in a significant increase in kinetochore volume. Data shown as the mean ± SD after 3D reconstruction of wild-type (150) and cKO (*n* = 141) kinetochores in three experimental replicates with *P* < 0.0001 (unpaired *t*-test, two tailed). Scale bar = 2 μm. **c** *Lsh-cKO* preovulatory oocytes exhibit fragmented kinetochores (see inset in (**b**)) and abnormal kinetochore fusions between different chromosome bivalents at the metaphase-I stage ((**c**); arrowhead and center diagram). Notably, abnormal kinetochore interactions between bivalent chromosomes result in the formation of ectopic kinetochores as indicated by the presence of metaphase-I bivalents with three CREST signals (arrowheads and right diagram). Scale bar (2 μm). *Lsh* conditional deletion induced a significant increase in the proportion of oocytes with kinetochore defects. Data represent the mean ± SD of three independent replicates with wild-type (*n* = 10) and cKO (*n* = 20) oocytes. Fused: *P* < 0.0468; and fragmented: *P* < 0.0042 using unpaired *t*-tests, two tailed.

H3T3ph is required to establish the inner centromere in both yeast and human cells^8^. In HeLa cells, H3T3ph is critical to coordinate sister chromatid cohesion with chromosome bi-orientation^8^. However, the mechanisms regulating chromatin modifications at the inner centromere remain to be established. In wild-type oocytes, H3T3ph decorates the inter-chromatid axis of M-I chromosomes and, as expected, fused sister kinetochores are detected on each bivalent (Fig. 6a, upper panel). By contrast, and consistent with our results using prophase-I oocytes, >80% of mutant preovulatory oocytes exhibit a striking increase in H3T3 phosphorylation both at M-I and M-II chromosomes. This was also confirmed by western blot analysis (Fig. 6a). Structured illumination revealed that sister kinetochores in mutant oocytes fail to fuse at M-I and frequently fragment and stretch into fibers that lack higher order structure resulting in a significant increase in kinetochore volume (Fig. 6b; insets and Supplementary Movies 6 and 7). Importantly, loss of proper kinetochore structure and fragmented kinetochores in >70% of mutant oocytes resulted in the formation of abnormal fusions between the centromere–kinetochore domains of two different M-I bivalents (Fig. 6c; arrowhead and center diagram). Strikingly, we found evidence for the formation of M-I bivalents with ectopic kinetochore localization in mutant oocytes indicated by the presence of stretched chromatin fibers labeled with CREST at distal chromosomal regions (Fig. 6c; arrowhead). These results indicate that LSH plays either an indirect or direct role in regulating the levels and chromosomal localization of H3T3ph in preovulatory oocytes and is critical for kinetochore structure and function. Importantly, our results provide the first evidence indicating that loss of LSH results in abnormal centromere–kinetochore fusions between different M-I bivalents and ectopic kinetochore formation suggesting the presence of deleterious chromosomal exchanges proximal to the centromere and chromosome nondisjunction.

**Fig. 7:**
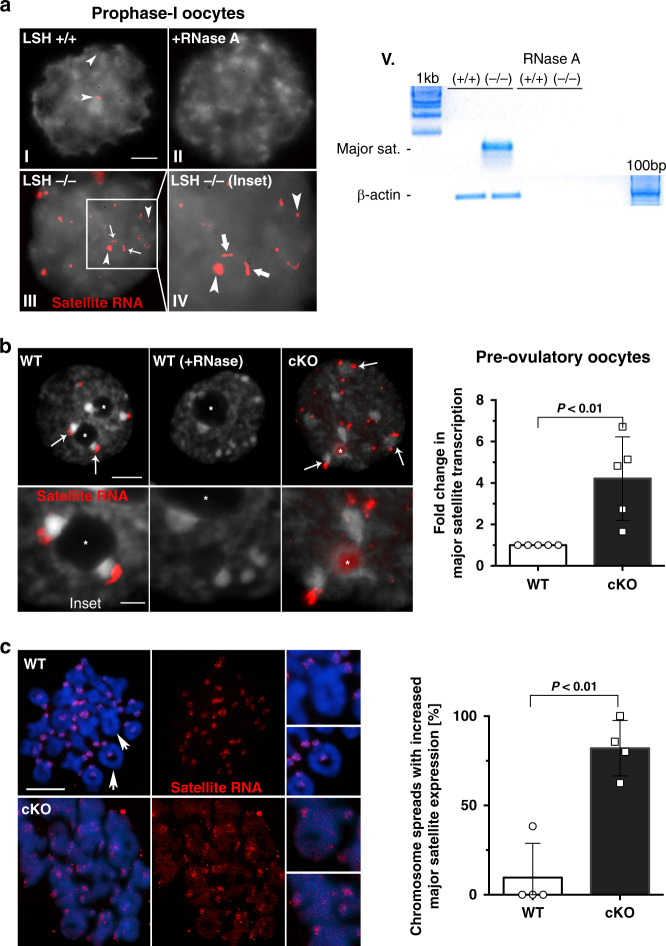
LSH conditional knockout induces overexpression of centromeric noncoding RNAs. **a** LSH is required for the epigenetic silencing of major satellite transcripts at key stages of oocyte meiosis. I RNA-FISH in wild-type oocytes indicates that major satellite transcripts (red; arrowheads) are expressed, albeit at low levels, and remain chromatin bound during meiotic prophase-I. II No signal was detected in the negative controls after exposure of oocyte nuclei to RNase A, confirming the presence of single stranded RNA. However, LSH^(−/−)^ oocytes exhibit a striking overexpression of centromeric noncoding RNAs. III, IV In situ detection of centromeric transcripts by RNA-FISH in mutant oocytes revealed the formation of tracks of chromatin bound centromeric RNA (arrows) as well as foci of different size (arrowheads). Scale bar (5 μm). V Major satellite transcripts are undetectable by qPCR in the fetal ovaries of wild-type littermates. Notably, they are overexpressed in LSH mutant littermates on day E17.5. **b** Major satellite transcripts (red) accumulate and remain associated with the large chromocenters (arrows) of wild-type preovulatory oocytes that exhibit the non-surrounded nucleolus configuration (inset). No signal was detected after the treatment of oocyte nuclei with RNAse. Notably, the loss of LSH function results in overexpression of centromeric transcripts and accumulation throughout the nucleoplasm as well as the nucleolus (inset). The position of the nucleolus is indicated by (*). Scale bar (10 μm). Inset scale bar (3 μm). Consistent with RNA-FISH, real-time PCR revealed a fourfold increase in the levels of major satellite transcripts in Lsh^−/^^−^ oocytes. Data are expressed as the mean ± SD of five independent experimental replicates with (*n* = 50) wild-type and (n = 50) cKO oocytes per replicate. Statistical analysis was performed using unpaired *t*-tests, two tailed with *P* = 0.0073. **c** Major satellite transcripts (red) remain associated with pericentric heterochromatin domains of metaphase-I chromosome bivalents in wild-type oocytes. Notably, loss of LSH function results in overexpression of satellite RNA that remains associated as multiple foci in metaphase-I bivalents. *Lsh*-cKO oocytes exhibit a significant increase in the proportion of chromosome spreads with elevated major satellite expression. Data are expressed as the mean ± SD of four independent experimental replicates with (*n* = 37) wild-type and (*n* = 29) cKO oocytes per replicate. Scale bar (10 μm). Statistical analysis was performed using unpaired *t*-tests, two tailed with *P* = 0.0011.

**Fig. 8:**
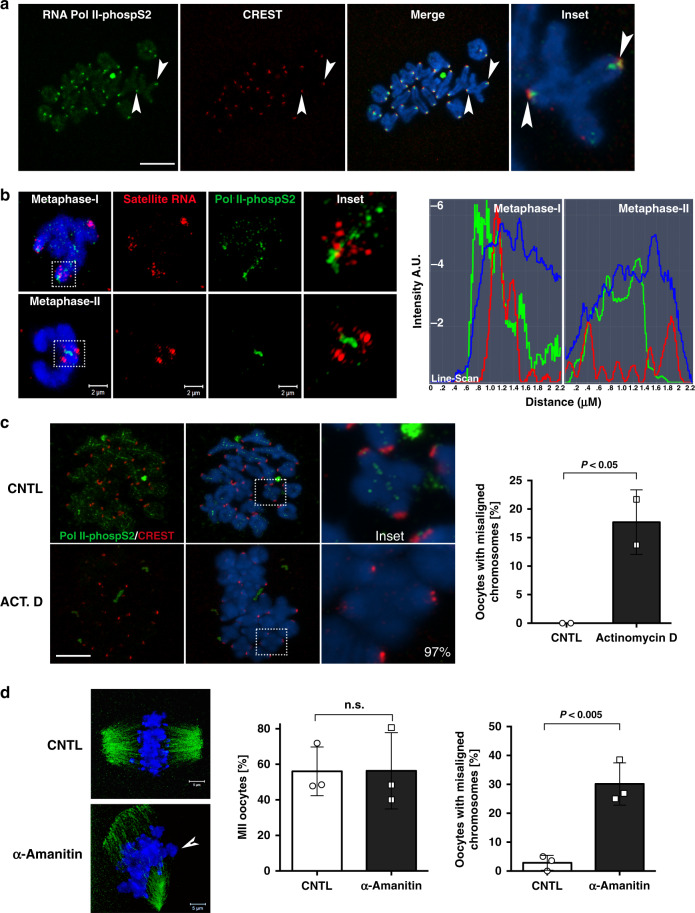
Co-localization of major satellite RNA with RNA polymerase-II (Pol II-phospS2) in meiotic chromosomes. **a** RNA polymerase-II phosphorylated at serine 2 (Pol II-phospS2; green) is present at the kinetochore of metaphase-I bivalents where it is co-localized with CREST signals (red). Scale bar = 10 μm. Pol II-phospS2 also extends to the inter-chromatid space (inset). **b** Super-resolution structured illumination revealed that satellite RNA foci (red) remain localized to the pericentric heterochromatin domains of metaphase-I chromosomes where several foci partially co-localize with Pol II-phosp2 (green) staining at the kinetochore. Extension of Pol II-phosp2 into the inter-chromatid space results in a linear staining pattern (inset). Line-scan analysis along the inter-chromatid axis reveals the extent of co-localization at metaphase-I. In metaphase-II chromosomes, Pol II-phospS2 is localized to the inner centromere, while satellite RNA foci appear as two flanking signals with limited co-localization with Pol II-phospS2 along the inter-chromatid axis (inset and line scan). **c** Exposure of oocytes to actinomycin D (1 μg/ml/1 h) interferes with the localization of Pol II-phospS2 to meiotic chromosomes in the majority of oocytes (lower inset), suggesting that this process is dependent on active transcription. Data are expressed as the mean ± SD of two experimental replicates with control (*n* = 37) and treated (*n* = 32) chromosome spreads. Statistical analysis was performed using unpaired *t*-tests, two tailed with *P* = 0.0475. Scale bar = 10 μm. Importantly, exposure to actinomycin D results in a significant increase in the proportion of oocytes that exhibit misaligned chromosomes at the meiotic spindle. Data from two independent experimental replicates with control (*n* = 42) and treated (*n* = 44) oocytes. **d** Exposure to a second transcriptional inhibitor, α-Amanitin (50 μg/ml) for 17 h, had no effect on the proportion of oocytes reaching the metaphase-II stage. However, it induced a significant increase in the proportion of in vitro matured oocytes with abnormal chromosome alignment to the meiotic spindle (arrowhead). Data are presented as the mean ± SD from three experimental replicates with control (*n* = 79) and treated (*n* = 77) oocytes. Statistical analysis was performed using unpaired *t*-tests, two tailed with *P* = 0.9864 (M-II oocytes) and *P* = 0.0037 (misalignment).

The presence of mirror structural defects at centromere–kinetochore domains in both prophase-I and M-I mutant oocytes prompted us to interrogate the mechanisms by which LSH may contribute to the epigenetic control of meiotic centromere function. In somatic cells, centromeric transcription during mitosis is an important mechanism regulating both kinetochore function and the chromosomal localization of distinct epigenetic marks required for pericentric heterochromatin formation^24–26^. However, whether centromeric transcription occurs in mammalian meiotic chromosomes remains to be demonstrated. Therefore, we used RNA-FISH to interrogate the expression and sub-chromosomal localization of major satellite transcripts at key stages of oocyte meiosis. In wild-type oocytes at prophase-I, centromeric transcripts are expressed at low levels but consistently detected in the nucleus of pachytene oocytes (Fig. 7a). Importantly, treatment with RNAse A eliminated all hybridization signals confirming the presence of single stranded RNA transcripts. By contrast, increased transcription of major satellites in LSH mutant oocytes resulted in the formation of tracks of centromeric RNA that accumulate in the nucleus of pachytene oocytes (Fig. 7a; inset). Consistent with their low expression, centromeric transcripts are undetectable by real-time PCR in wild-type fetal ovaries on day E17.5 but are significantly upregulated in LSH mutant gonads (Fig. 7a). RNA-FISH revealed that major satellite transcripts are abundant in wild-type preovulatory oocytes at the GV stage where centromeric RNA expression leads to the formation of a single, dense cloud capping the largest chromocenters in the oocyte nucleus. Treatment with RNAse A removed all hybridization signals indicating the presence of single stranded RNA (Fig. 7b). Importantly, *Lsh*^(−/−)^ oocytes exhibited a prominent accumulation of multiple fragmented foci of RNA signals mostly associated with chromocenters but also detectable in the nucleoplasm as well as the nucleolus (Fig. 7b). Quantification of major satellite transcripts by qPCR revealed a fourfold increase in centromeric transcript expression in LSH mutant oocytes (Fig. 7b). These results indicate that centromeric major satellite transcripts are expressed at key stages of oocyte meiosis and that LSH plays a critical role in regulating their levels of expression and nuclear distribution. Therefore, we set out to determine the chromosomal fate of centromeric RNA during oocyte meiotic maturation. Remarkably, RNA-FISH revealed that centromeric transcripts are detected to different extent at all centromeres of M-I chromosome bivalents in wild-type oocytes (Fig. 7c). Centromeric transcripts remain exclusively associated with pericentric heterochromatin domains detected by bright DAPI staining in wild-type oocyte bivalents at the M-I, whereas in LSH mutant oocytes, satellite RNA overexpression leads to the formation of multiple foci in addition to the bright centromeric localization in 82.1% of chromosome spreads analyzed (Fig. 7c; lower panel). To determine whether centromeric RNAs in meiotic chromosomes require polymerase-II-dependent transcriptional regulation, we analyzed the sub-chromosomal localization of RNA polymerase-II phosphorylated at the Serine 2 residue (Pol II-phospS2), which is required for transcriptional elongation^25^. Remarkably, Pol II-phospS2 (green) is present at the kinetochore and inner centromeres of all M-I bivalents where it is co-localized with CREST signals (red) (Fig. 8a; inset). Super-resolution and line-scan analysis revealed that Pol II-phosp2 foci (green) may extend into the inter-chromatid space of M-I bivalents where it is partially co-localized with satellite RNA foci (red) that accumulate at pericentric heterochromatin (Fig. 8b; top inset). In addition, Pol II-phosp2 is present at the inner centromere of M-II stage chromosomes where, it is flanked by satellite RNA foci (Fig. 8b; lower inset). These patterns of meiotic localization are dependent on transcription as indicated by the loss of Pol II-phospS2 staining in 97% of oocytes after 1-h exposure to the transcriptional inhibitor actinomycin D (Fig. 8c; inset), while no changes were observed in the inter-chromatid localization of the cohesin protein SMC3 used as a negative control (Supplementary Fig. 5). Notably, a brief exposure to actinomycin D or treatment with α-Amanitin throughout the entire maturation period interfered with accurate chromosome alignment to the meiotic spindle in 17.7% and 30.1% of M-II stage oocytes, respectively (Fig. 8c, d). No significant differences were observed in the proportion of M-II stage oocytes matured in the presence or absence of α-Amanitin. However, the high incidence of misaligned chromosomes suggests a function for centromeric transcripts in the regulation of chromosome–microtubule interactions. Notably, microinjection of validated locked nucleic acid (LNA) gapmers Maj1/2 (ref. ^27^) to knockdown major satellite RNAs at the GV stage induced abnormal chromatin conformation in ~60% of oocytes (Supplementary Fig. 6). These results indicate that major satellite transcripts are expressed at the meiotic centromere and that LSH plays a critical role in regulating their levels and chromosomal localization during oocyte meiosis.

## Discussion

LSH is a major regulator of DNA methylation at repetitive sequences in the mouse genome^12,13^. Notably, a missense mutation in human LSH has been recently identified in patients with immunodeficiency, centromere instability, and facial anomaly syndrome^28^. However, the role of LSH in centromere function remained unexplored. Here, we uncover a noncanonical role for LSH in meiotic kinetochore function, transcriptional silencing of centromeric noncoding RNAs, and regulation of both, the levels and chromosomal localization of H3T3 phosphorylation. Although LSH mutant oocytes exhibit no significant differences in the levels of H3K9me3 (ref. ^16^), super-resolution analysis indicates that LSH is essential for meiotic centromere compaction and structural integrity. Our results provide novel evidence indicating that LSH is present at the meiotic kinetochore where it functions to regulate cytosine methylation (5-mC) at pericentric heterochromatin as well as the levels of H3T3ph required for centromere function and accurate chromosome segregation during female meiosis.

LSH exhibits a sexually dimorphic and remarkably dynamic subcellular localization in male and female germ cells. In pachytene oocytes, it is a prominent nucleoplasmic protein and targeted deletion results in reduced HDAC2 and DNMT-1. Notably by decreasing the levels of CDYL, a repressor of histone crotonylation^22^ and increasing transcripts for the histone acetyl transferase MYST4 (ref. ^29^), *Lsh* deletion may result in a transcriptionally permissive chromatin environment in mutant oocytes. Biochemical assays indicate that LSH interacts with both DNMT-1 and HDAC2 when targeted to the promoter of a reporter gene in vitro^30^. Our studies revealed that LSH exhibits a functional interaction with these key components of the global DNA methylation complex in vivo, where it might be required to recruit the DNA methylation machinery to tandem repeats in pachytene oocytes as demonstrated by the loss of 5-mC staining at pericentric heterochromatin. These results also provide a mechanistic explanation for the loss of DNA methylation at major and minor satellite DNA sequences previously observed in prophase-I oocytes^16^. The molecular mechanisms responsible for increased H3/Thr3 phosphorylation in mutant oocytes remain to be established. In somatic cells, Haspin kinase regulates H3/Thr3 phosphorylation^8,9^. Notably, Haspin gene expression is regulated by DNA methylation at a specific differentially methylated region (DMR) upstream of its gene promoter. This unique DMR acts as a *cis*-regulatory element that is highly methylated in the ovary^31^.Thus, abnormal DNA methylation at specific loci in LSH mutant oocytes may affect Haspin gene expression leading to increased H3T3ph levels. Alternatively, abnormal histone modifications brought about by loss of LSH function may also affect the levels of H3/Thr3 phosphorylation. Previous studies indicate that the epigenetic context of a chromosomal domain may also affect the levels of H3/Thr3 phosphorylation^32,33^. For example, the juxtaposition of H3K4me at an amino acid adjacent to H3T3 is known to change the levels of H3/Thr3 phosphorylation in fragment peptides in vitro^32,33^, and loss of LSH function disrupts the levels and genomic distribution of H3K4me1 in mouse somatic cells^14,15^. Experiments are in progress to distinguish among these possibilities. Both mechanisms, however, suggest an epigenetic control indicating that LSH may be at the center of a regulatory pathway controlling a functional crosstalk between DNA methylation and histone H3/Thr3 phosphorylation to maintain chromosome stability during female meiosis.

In human and mouse somatic cells, centromeric noncoding RNAs have emerged as critical epigenetic determinants of heterochromatin formation and kinetochore function^24–26^. Transcription of centromeric RNAs is under a strict spatial and temporal control during mouse development^34^ but their presence and function during mammalian meiosis remained to be demonstrated. Our results provide the first evidence for the expression of major satellite transcripts at key stages of oocyte meiosis and a potential role in the regulation of chromosome alignment to the meiotic spindle. Importantly, we demonstrate that LSH is required to regulate centromeric transcripts. In addition to its nucleoplasmic localization, LSH is enriched at the inner centromere in late pachytene, and targeted deletion-induced abnormal kinetochore structure, centromere fusions, and striking ectopic kinetochore formation suggesting the presence of abnormal chromosome exchanges proximal to the centromere. In model organisms, such as the fungus *Ascobolus*, the loss of DNA methylation results in aberrant meiotic recombination. Notably, lack of CG methylation and transcriptional reactivation of centromeric repeats increase the formation of centromere proximal crossovers in *Arabidopsis thaliana*^35,36^. Our results indicate that LSH is required for kinetochore function and maintenance of a transcriptionally repressive heterochromatin environment to prevent abnormal centromere interactions that may predispose to harmful recombination of centromeric tandem repeats. Importantly, elegant studies have recently identified a novel, meiosis-specific role for the kinetochore in preventing the formation of potentially deleterious centromere proximal crossovers in budding yeast^37^.

LSH is present both at the inner centromere and the kinetochore of M-I oocytes. Notably, and consistent with a prominent role in meiotic kinetochore function and centromeric transcription at key stages of meiosis, conditional deletion in preovulatory oocytes induced mirror defects as those observed in mutant prophase-I oocytes, including increased H3T3 phosphorylation, abnormal kinetochore structure, fusions between the kinetochores of adjacent M-I chromosome bivalents and ectopic kinetochores. Histone H3T3 phosphorylation is critical for centromere function in mitotic cells^8^. However, the mechanisms regulating specific chromatin modifications at the inner centromere are not known. Our results indicate that LSH is present both at the meiotic kinetochore and inner centromere where it is required to regulate the levels of H3T3ph in mammalian oocytes. In somatic cells, H3T3ph acts as a transient off switch that ejects chromatin-modifying proteins from the centromeres during mitosis^38^. However, overexpression of H3T3ph or inappropriate persistence at the centromere during anaphase delays mitotic progression^39,40^. Notably, abnormal targeting of H3T3ph to the kinetochore results in chromosome instability indicative of centromeric dysfunction such as Robertsonian (centromere) fusions and trisomies in human ES cells^38^.

In contrast to prophase-I oocytes, centromere major satellite transcripts accumulate at most chromocenters of wild-type preovulatory oocytes at the GV stage. Our results provide the first evidence indicating that both satellite RNA and the transcriptionally engaged isoform of RNA polymerase-II (Pol II-phospS2) remain at the centromeres of M-I chromosomes, suggesting that centromeric noncoding RNAs associate with a marker of active transcription at the meiotic centromere. Importantly, LSH conditional deletion induced a fourfold increase in the levels of major satellite transcripts as well as an accumulation of satellite RNA throughout the chromatids of M-I chromosomes. Overexpression of noncoding centromeric transcripts may lead to abnormal localization of key kinetochore proteins and abnormal centromere function in mouse somatic cells^24,26^. We propose that by regulating DNA methylation at pericentric heterochromatin and maintaining transcriptional silencing of major satellite sequences, LSH impacts centromere and kinetochore function. In turn, loss of transcriptionally repressive heterochromatin formation may predispose to abnormal centromeric recombination events, kinetochore fusions, and chromosome nondisjunction.

Consistent with this notion, loss of LSH function in preovulatory oocytes delayed the resumption of meiosis and resulted in abnormal chromosome segregation, leading to significantly increased rates of in vitro M-I arrest in mutant oocytes and a significant reduction in female fertility. In addition to its kinetochore localization during oocyte maturation, LSH exhibits a remarkable redistribution to the mid-body at anaphase and to the spindle poles at M-II, suggesting that LSH integrates kinetochore structure with spindle function and chromosome segregation. Loss of LSH disrupts nucleosome organization at heterochromatin repeat sequences in mouse ES cells^14^ and both lack of nucleosome formation and point mutations on H3T3ph result in abnormal spindle assembly^10^. The molecular mechanisms leading to abnormal chromosome segregation remain to be determined. However, increased H3T3 phosphorylation in mutant oocytes may interfere with anaphase-I onset by preventing the timely release of sister chromatid arm cohesion or by disrupting accurate bivalent chromosome bi-orientation. Current studies are aimed at distinguishing among these possibilities.

Both abnormal meiotic recombination and nondisjunction are critical components of a two-hit mechanism resulting in oocyte chromosome aneuploidy^6^. Notably, in human oocytes, inefficient maturation of crossovers generates chromosome interactions that are prone to missegregation and aneuploidy^41^. Centromere proximal crossovers are associated with an increased risk of nondisjunction and human trisomies^6^. Since the initial description of the centromere effect in *Drosophila,* the critical role of pericentric heterochromatin formation in preventing the formation of crossovers near the centromere has been well established in several species including human chromosomes^42,43^. However, the molecular mechanisms are poorly understood. Our studies provide novel insight into this critical mechanism of mammalian genome stability, suggest a role for LSH in this process, and inform the mechanisms by which disruption of chromatin marks at the centromere may result in abnormal chromosome segregation and oocyte aneuploidy. Collectively, our results demonstrate a novel, noncanonical role for LSH in kinetochore function, centromere stability, and chromosome segregation during female meiosis. Further analysis of LSH function will be essential to determine its specific roles in the long-sought mechanisms regulating the centromere effect and in preventing chromosome nondisjunction during female meiosis.

## Methods

### Animal models

This study was approved by and performed in accordance with guidelines of the University of Georgia Institutional Animal Care and Use Committee. Conventional knockout (LSH^−/^^−^) fetuses were obtained from timed matings between heterozygote Hells^tm1Kmu^ mice as described previously^16^. Conditional knockout (LSH-cKO) females were generated by crossing C57BL/6NTac-Hellstm1a(EUCOMM)Wtsi/leg mice (European Conditional Mouse Mutagenesis Program), carrying a knockout first allele for tissue-specific targeting of LSH, with Act-FLPe mice (B6;SJL-Tg(ACTFLPe)9205Dym/J) first to remove the reporter cassette via FLP-FRT recombination. The following genotyping primers were used for this step in addition to those recommended by EUCOMM for tm1c conversion: LSH-flip-1-fwd 5′-CGG AAA ACA GTA TCT TCA GGA TGG A-3′, and LSH-flip-1-rev 5′-CAG GCC AGC TTG ACT CAA AT-3′. Offspring with floxed Lsh alleles was subsequently crossed with Zp3-Cre transgenic mice (C57BL/6-Tg(Zp3-cre)93Knw/J) to induce inactivation of maternally expressed LSH in growing and fully grown oocytes by means of deletion of Exon 11, inducing a frame shift mutation and a premature STOP codon. The following primer sequences were used to distinguish different genotypes: presence of Cre recombinase: CRE-Fwd 5′-CAT TTG GGC CAG CTA AAC AT-3′, CRE-Rev 5′-TAA GCA ATC CCC AGA AAT GC-3′; successful tm1d conversion/loxP recombination: tm1d-Fwd 5′-AAG GCG CAT AAC GAT ACC AC-3′, tm1d-Rev 5′-ACT GAT GGC GAG CTC AGA CC-3′, LoxP-Fwd 5′-ATC CGG GGG TAC CGC GTC GAG-3′, LoxP-Rev’ 5′-ACT GAT GGC GAG CTC AGA CC-3′; and loss of Exon 11: lossE11-Fwd 5′-AAG TGC CGG CTA ATC AGG GA-3′, lossE11-Rev 5′-AGG ACA TGT ATG CTG CCT GG-3′.

For fertility analyses, wild-type and LSH-cKO females 2 months of age were housed continuously with age-matched wild-type males of proven fertility (1:1 ratio) for a period of 6 months. Fertility parameters, such as litter size and parturition frequency, were monitored.

### Oocyte collection and culture

Fetal ovaries were recovered on 18 dpc and immediately processed for in situ analyses or flash-frozen and stored at −80 °C. Fully grown GV stage oocytes were harvested from ovaries of 21–23-day-old mice as described previously^18^. Oocytes at the GV stage and those undergoing GVBD, as well as at pro-M-I and the M-I stage were collected following a culture of 0, 1.5, 2, 4, 6, and 8 h, respectively. The in vitro maturation potential was assessed at 16 h post release from milrinone (Sigma; 1 µg/ml) arrest in control and cKO oocytes. M-II eggs were obtained 13–15 h after superovulation with hCG (EMD Biosciences) according to standard protocols.

Denuded oocytes were fixed in 2% paraformaldehyde in PBS for 20 min, and permeabilized with 1% Triton X at room temperature for 20 min. The oocytes were then washed and blocked in PBS supplemented with 5% serum and 0.05% Triton X-100. Preparation of surface-spread fetal oocytes was conducted as previously described^16^ with the following modifications. Upon hypotonic treatment for 25 min, fetal oocytes were dried down in a fixative solution of 2% PFA, 0.1% Triton X-100 in nuclease-free water on glass slides for 30 min prior to storage at −80 °C. This improved fixing protocol is critical for enhanced epitope accessibility on condensed chromatin, while simultaneously preserving kinetochore structure and analysis of chromatin bound proteins^18^. Metaphase chromosome spreads were prepared from zona-free oocytes as described previously^18^. Briefly, the zona pellucida was removed with Tyrode’s solution (Sigma) before recovery in culture medium for 5 min. Chromosomal proteins were cross-linked by spreading the oocytes on a wet slide containing 1% paraformaldehyde and 0.15% Triton X in nuclease-free water. Slides were allowed to dry at room temperature and stored at −80 °C. For the analysis of meiotic spindle poles, residual fixative was carefully aspirated from the periphery of the drop to lyse oocytes and simultaneously preserve the meiotic spindle apparatus. Slides were air dried without disturbance and washed once in PBST (PBS containing 0.05% Tween-20) for 5 min before immunochemistry.

### Treatment with transcriptional inhibitors

Cumulus-oocyte complexes were collected from the ovary and cultured in MEM/BSA medium, supplemented with 5% FBS and 50 µg/ml alpha-amanitin (Sigma) for 17 h. In vitro matured oocytes were the fixed in 4% PFA, 0.05% Tx-100 for 30 min at 37 °C. Oocytes were washed in PBS, 5% FBS, 0.01% Tx-100 three times before blocking. Oocytes were then stained with mouse acetylated-tubulin antibody as described below. Actinomycin D (1 µg/ml) was added to the treatment group for the final hour of a 16 h IVM incubation in MEM/BSA + 5% FBS.

### Immunocytochemistry

Unless indicated otherwise, all primary antibody incubations were conducted in block buffer (PBS, 5% serum, 0.05% Triton X-100) overnight at 4 °C. All antibodies are listed in Supplementary Table 1. Meiotic configuration in wild-type and mutant oocytes at the pachytene stage of meiosis was determined by immunochemical detection of the lateral elements of the SC using a 1:500 dilution of a polyclonal mouse anti-SYCP3 antibody (abcam). The subcellular localization of LSH protein in wild-type oocytes at prophase-I, the GV stage, and at M-II of meiosis was determined using a 1:500 dilution of a rabbit anti-LSH antibody (anti-Smarca6, abcam). Centromeres were labeled with a 1:500 dilution of anti-CREST antiserum (Nuclear ANA-Centromere Autoantibody, Cortex Biochem). Kinetochore domains were labeled using CENP-A (1:500, Cell Signaling Technologies) and CENP-C (1:200, a generous gift from Bill Earnshaw) antibodies. An anti-phospho-histone H3T3 rabbit antibody (EMD Millipore) was used at 1:400. The following antibodies were diluted 1:200 prior to incubation: rabbit anti-HDAC2 (abcam), rabbit anti-DNMT-1 (pATH52, a generous gift from Tim Bestor), mouse anti-5-mC (Calbiochem), mouse anti-Pol II phospho Ser2 (Abcam), rabbit anti-SMC3 (abcam). Mouse anti-acetylated tubulin (Sigma) was used at 1:1000 for 1 h at 37 °C. Spindle poles were detected with an anti-mouse Pericentrin antibody (1:400). Immunodetection was performed using appropriate combinations of Alexa Fluor-488 or 555-conjugated IgG Fab fragments as secondary antibodies (Molecular Probes) at a dilution of 1:1000 for 1 h at room temperature. Samples were counterstained in mounting medium containing DAPI (4′, 6-diamidino-2-phenylindole; Vectashield plus DAPI, Vector Laboratories).

### Proximity ligation assays (PLA)

In situ detection of endogenous DNMT-1 protein in fetal oocytes by quantitative proximity ligation was conducted according to manufacturer’s instructions (Sigma) using an anti-rabbit DNMT-1 primary antibody (pATH52, a generous gift from Tim Bestor). Briefly, following overnight incubation of the samples with DNMT-1 antibody and extensive washes, plus and minus anti-rabbit PLA probes were used to bind primary antibody and connector oligos were hybridized. Ligation enabled the formation of circular DNA molecules required for subsequence rolling-circle amplification by DNA polymerase. The resulting concatemeric sequences were detected following hybridization of complementary fluorescently labeled detection oligos that were visualized and quantified as discrete PLA signals by fluorescence microscopy analysis.

### Western blotting

Sample preparation for western blotting was conducted as described^44^. Briefly, denuded oocytes (*n* = 50) were collected and frozen in Laemmli buffer with protease inhibitors (Sigma). Prior to gel electrophoresis, samples were boiled at 100 °C for 5 min and then separated in 7.5% acrylamide gels containing 0.1% SDS. Proteins were transferred onto hydrophobic PVDF membranes (Amersham). Membranes were blocked with 5% non-fat milk in PBST (PBS with 0.1% Tween-20) overnight at 4 °C and then incubated with rabbit anti-LSH antibody (a gift from Dr. Kathrin Muegge, National Cancer Institute, 1/5000 in PBST) or anti-H3T3ph (EMD Millipore, 1:1000 dilution) at 4 °C overnight and subsequently washed in PBST. After three washes in PBST for a total of 60 min, the membranes were labeled with peroxidase-conjugated goat anti-rabbit IgG (Invitrogen, 1/5000 in PBST) for 1 h. The proteins were visualized using an ECL-plus detection system (Amersham). Some membranes were also probed with anti-β tubulin (Sigma Aldrich, 1/2000 dilution) as an internal control. Uncropped/unprocessed scans of all western blots can be found in the Source Data file.

### RNA fluorescence in situ hybridization (RNA-FISH)

RNA-FISH experiments were conducted essentially identical to previously described procedures^18^. Briefly, fetal oocytes and zona-free GV stage or M-I oocytes were permeabilized with a solution of 0.5% Triton X-100 in DEPC-PBS for 2 min at room temperature and washed three times in 2xSSC. Control spreads were treated with 100 µg/ml RNase A (Roche, Basel, Switzerland) for 1 h at 37 °C and subsequently washed extensively in 2xSSC. A major satellite-specific FISH probe (Cambio Ltd) was denatured at 80 °C for 8 min, and then cooled to 37 °C before application onto the surface-spread samples. Hybridization was carried out overnight at 37 °C in a humidified chamber prior to two post-hybridization washes in 1xSSC in DEPC-H_2_O for 5 min at room temperature and mounting in Vectashield plus DAPI.

### RNA isolation, qPCR, and PCR arrays

Pathway-focused gene expression analyses were conducted using mouse PCR Arrays for Epigenetic Chromatin-Modifying Enzymes (SABiosciences; QIAGEN). This allowed the simultaneous quantification of a panel of 84 mRNA transcripts encoding for key chromatin-modifying enzymes known to establish genomic DNA methylation as well as enzymes that regulate essential histone posttranslational modifications including histone acetylation, methylation, phosphorylation, and ubiquitination. mRNA was isolated from flash-frozen wild-type (*n* = 3) and LSH knockout (*n* = 3) fetal ovaries obtained from timed matings on 18 dpc using the Micro-FastTrack 2.0 mRNA isolation kit (Invitrogen) as described before^45^. RT efficiency, qPCR performance, and cDNA quality were confirmed in array-integrate control reactions. Expression data were normalized to a series of internal housekeeping genes and data points were excluded from the analysis when expression levels of genes were below detectable limits. Raw threshold cycle data between wild-type ovaries and LSH^−^^/−^ ovaries were compared using RT^2^ Profiler PCR Array Data Analysis software (SABiosciences; QIAGEN) version 3.5 to conduct all ΔΔt-based fold-change calculations.

For qPCR assays to analyze residual Lsh expression in cKO oocytes, mRNA was isolated from groups (*n* = 50) of denuded oocytes using the miRNeasy kit (Qiagen) and subsequently reverse transcribed according to manufacturer’s instructions to assess transcript levels of Lsh using a validated RT^2^ qPCR Primer Assay (SABiosciences). qPCR results were normalized against Gapdh (RT^2^ qPCR Primer assay, SABiosciences) transcript levels as a housekeeping control.

Major satellite expression was conducted on total RNA extracted from fetal ovaries and denuded fully grown oocytes using random hexamer primers and the Superscript II first strand synthesis system (Invitrogen) before semi-quantitative or qPCR using the RT^2^ SYBR Green PCR Master Mix (Qiagen) on a RotorGene Q100 light cycler apparatus (Qiagen). Raw threshold cycle data obtained from wild-type and LSH^−/−^ oocyte samples were analyzed using RT^2^ Profiler PCR Array Data Analysis software version 3.5 (SABiosciences; Qiagen) to conduct all ΔΔCt-based calculations. Primer sequences were described previously^46^. Major satellite expression levels were normalized using beta-actin transcripts as normalizer (Beta-actin-Fwd 5′*-*ggc acc aca cct tct aca atg*-*3′; Beta-actin-Rev 5′*-*gtg gtg gtg aag ctg tag cc-3′).

### Microinjection

To assess LSH function during resumption of meiosis in mouse oocytes, capped mRNAs encoding fluorescently labeled histone H2B-fusion proteins (H2B-GFP and H2B-RFP) were in vitro transcribed from plasmids pGEMHE-H2B-RFP (Euroscarf) and pIVT-H2B-GFP (a generous gift from Dr. R Schultz), respectively, as described previously^44^ and microinjected into the cytoplasm of oocytes 15–18 h prior to resumption. In brief, 10 pl capped mRNA of 10 µM stock solution were microinjected into the cytoplasm of GV-intact oocytes in MEM medium containing 1 µg/ml milrinone. Wild-type oocytes were microinjected with H2B-GFP capped mRNA. LSH-cKO oocytes received capped mRNA encoding H2B-RFP. Following overnight incubation to allow for expression of fusion proteins, oocytes were washed and transferred to a micro drop of fresh medium (without milrinone) supplemented with 5% FBS under mineral oil. Resumption of meiosis was monitored in real time by live-cell imaging and confocal microscopy in an environmental chamber with an atmosphere of 5% CO2, 5% O2, and 90% N2.

For knockdown of major satellite transcripts, GV stage oocytes were collected from non-primed female mice of a C57BL/6/DBA hybrid background, denuded and microinjected with 10 pl of a 10 µM cocktail of forward and reverse LNA-DNA major satellite gapmers in 20 mM KCl, 0.2 mM MgCl_2_ using an Eppendorf micromanipulator on a Nikon inverted microscope. Control oocytes were in parallel injected with LNA-DNA GFP gapmers. Oocytes were then cultured for 24 h to allow for the knockdown of transcripts, before fixing in 4% PFA, 0.05% Tx-100 for 30 min at 37 °C. Chromatin was stained with an anti-histone H2B nanobody (1:200; ChromoTek). Chromocenters were stained with an anti-ATRX antibody at 1:200 overnight at 4 °C before mounting on glass slides and microscopic analysis.

### Image acquisition, processing, and fluorescence quantification

Epifluorescence and RNA-FISH image acquisition was conducted using a Leica DMRX/E fluorescence microscope (Leica Microsystems, Inc.) equipped with a HCX PLAN APO 40x/0.85 air, and with a PLAN APO 63x/1.20 water objective. Images were captured with a Leica DFC 350F camera using Openlab software 3.1.7 (Improvision) and image processing was performed using Photoshop 2.0 (Adobe) for linear adjustments and cropping of fluorescent images. No gamma adjustments were made. SR-SIM was performed on an Zeiss ELYRA S1 Super-resolution Microscope on an Axio Observer Z1 inverted microscope stand equipped with three laser lines: 405, 488, and 561 nm. Images were acquired using a ×100 objective and were processed with ZEN 2.3 software to generate maximum projections and SR-SIM computations.

Laser-scanning confocal microscopy and oocyte live-cell imaging was conducted as follows: 3D stacks were acquired every 30 min for 16 h using a Nikon Eclipse Ti-U/D-Eclipse C1 laser-scanning confocal microscope equipped with a ×40 objective lens following sequential (frame lambda) excitation of GFP fusion proteins with a 488 nm Coherent Sapphire laser and RFP fusion proteins with a 561 Coherent Sapphire laser. Image acquisition was conducted using EZC1 3.91 software (Nikon) with a step size of 5 μm and a Z-stack range of 100 μm. Live-cell imaging data were subsequently analyzed by maximum intensity and 3D reconstructions by using NIH Elements 4.0 software (Nikon) and the dynamics of key developmental stages of meiotic maturation were compared.

Fluorescence intensity quantification and morphological measurements were performed using the multidimensional imaging capabilities of the Automated Measurements Module of NIS Elements 4.0 software (Advances Research, Nikon Instruments) on confocal Z-stacks of wild-type and knockout samples imaged using identical laser power and gain parameters. Similar thresholds were established for all images before transformation to binary layers and automated conversion to regions of interest (ROIs). The average fluorescence intensity per unit area (in arbitrary units) within the ROI as well as size-morphometric parameters (e.g., surface area/volume) was recorded for each individual sample before final average values were calculated for all experimental groups/replicates.

### Statistics and reproducibility

Data are presented as the mean of at least three independent experiments; variation among replicates is presented as the standard deviation (SD). Comparison of all pairs was conducted using two-sided parametric and nonparametric tests (Mann–Whitney or unpaired *t*-test) according to the sample distribution (D’Agostino-Pearson) with GraphPad Prism 6 software. Differences were considered significant when *P* < 0.05 and *P* values are indicated. Box plot depict the median value as well as upper and lower quartiles with whiskers representing the minimum and maximum values observed. Dot plots show individual observations as dots as well as the mean with whiskers representing the SD.

### Reporting summary

Further information on research design is available in the Nature Research Reporting Summary linked to this article.

## Supplementary information

Supplementary Information

Description of Additional Supplementary Files

Supplementary Data 1

Supplementary Data 2

Supplementary Movie 1

Supplementary Movie 2

Supplementary Movie 3

Supplementary Movie 4

Supplementary Movie 5

Supplementary Movie 6

Supplementary Movie 7

Reporting Summary

## Data Availability

The authors declare that the data supporting the findings of this study are available within the paper and its supplementary information files (see Supplementary Data 1 and 2 and Source Data). All data are available from the authors upon reasonable request. Source data are provided with this paper.

## Source data

Source Data

## References

[1] Dillon N, Festenstein R (2002). Unravelling heterochromatin: competition between positive and negative factors regulates accessibility. Trends Genet..

[2] Ekwall K (2007). Epigenetic control of centromere behavior. Annu. Rev. Genet..

[3] De La Fuente R (2006). Chromatin modifications in the germinal vesicle (GV) of mammalian oocytes. Dev. Biol..

[4] Kimmins S, Sassone-Corsi P (2005). Chromatin remodelling and epigenetic features of germ cells. Nature.

[5] Petronczki M, Siomos M, Nasmyth K (2003). Un menage a quatre: the molecular biology of chromosome segregation in meiosis. Cell.

[6] Nagaoka SI, Hassold TJ, Hunt PA (2012). Human aneuploidy: mechanisms and new insights into an age-old problem. Nat. Rev. Genet..

[7] Cleveland D, Mao Y, Sullivan K (2003). Centromeres and kinetochores: from epigenetics to mitotic checkpoint signaling. Cell.

[8] Wang F (2010). Histone H3 Thr-3 phosphorylation by Haspin positions Aurora B at centromeres in mitosis. Science.

[9] Yamagishi Y, Honda T, Tanno Y, Watanabe Y (2010). Two histone marks establish the inner centromere and chromosome bi-orientation. Science.

[10] Zierhut C, Jenness C, Kimura H, Funabiki H (2014). Nucleosomal regulation of chromatin composition and nuclear assembly revealed by histone depletion. Nat. Struct. Mol. Biol..

[11] Ng TM (2013). Kinetochore function and chromosome segregation rely on critical residues in histones H3 and H4 in budding yeast. Genetics.

[12] Huang J (2004). Lsh, an epigenetic guardian of repetitive elements. Nucleic Acids Res..

[13] Tao Y (2011). Lsh, chromatin remodeling family member, modulates genome-wide cytosine methylation patterns at nonrepeat sequences. Proc. Natl Acad. Sci. USA.

[14] Ren J (2015). The ATP binding site of the chromatin remodeling homolog Lsh is required for nucleosome density and de novo DNA methylation at repeat sequences. Nucleic Acids Res..

[15] Yu W (2014). CG hypomethylation in Lsh−/− mouse embryonic fibroblasts is associated with de novo H3K4me1 formation and altered cellular plasticity. Proc. Natl Acad. Sci. USA.

[16] De La Fuente R (2006). Lsh, is required for meiotic chromosome synapsis and retrotransposon silencing in female germ cells. Nat. Cell Biol..

[17] Zeng W (2011). Lymphoid-specific helicase (HELLS) is essential for meiotic progression in mouse spermatocytes. Biol. Reprod..

[18] De La Fuente R, Baumann C, Viveiros MM (2015). ATRX contributes to epigenetic asymmetry and silencing of major satellite transcripts in the maternal genome of the mouse embryo. Development.

[19] Schermelleh L (2008). Subdiffraction multicolor imaging of the nuclear periphery with 3D structured illumination microscopy. Science.

[20] Wynne DJ, Funabiki H (2015). Kinetochore function is controlled by a phospho-dependent coexpansion of inner and outer components. J. Cell Biol..

[21] Soderberg O (2006). Direct observation of individual endogenous protein complexes in situ by proximity ligation. Nat. Methods.

[22] Liu S (2017). Chromodomain protein CDYL acts as a crotonyl-CoA hydratase to regulate histone crotonylation and spermatogenesis. Mol. Cell.

[23] Dennis K, Fan T, Geiman T, Yan Q, Muegge K (2001). Lsh, a member of the SNF2 family, is required for genome-wide methylation. Genes Dev..

[24] Bouzinba-Segard H, Guais A, Francastel C (2006). Accumulation of small murine minor satellite transcripts leads to impaired centromeric architecture and function. Proc. Natl Acad. Sci. USA.

[25] Chan FL (2012). Active transcription and essential role of RNA polymerase II at the centromere during mitosis. Proc. Natl Acad. Sci. USA.

[26] Velazquez Camacho O (2017). Major satellite repeat RNA stabilize heterochromatin retention of Suv39h enzymes by RNA-nucleosome association and RNA:DNA hybrid formation. eLife.

[27] Probst AV (2010). A strand-specific burst in transcription of pericentric satellites is required for chromocenter formation and early mouse development. Dev. Cell.

[28] Thijssen PE (2015). Mutations in CDCA7 and HELLS cause immunodeficiency-centromeric instability-facial anomalies syndrome. Nat. Commun..

[29] Klein BJ, Lalonde M-E, Côté J, Yang X-J, Kutateladze TG (2014). Crosstalk between epigenetic readers regulates the MOZ/MORF HAT complexes. Epigenetics.

[30] Myant K, Stancheva I (2008). LSH cooperates with DNA methyltransferases to repress transcription. Mol. Cell Biol..

[31] Sato S (2011). DNA methylation-dependent modulator of Gsg2/Haspin gene expression. J. Reprod. Dev..

[32] Han A (2011). Methylation-mediated control of aurora kinase B and Haspin with epigenetically modified histone H3 N-terminal peptides. Bioorg. Med. Chem..

[33] Eswaran J (2009). Structure and functional characterization of the atypical human kinase Haspin. Proc. Natl Acad. Sci. USA.

[34] Eymery A, Callanan M, Vourc’h C (2009). The secret message of heterochromatin: new insights into the mechanisms and function of centromeric and pericentric repeat sequence transcription. Int J. Dev. Biol..

[35] Maloisel L, Rossignol JL (1998). Suppression of crossing-over by DNA methylation in *Ascobolus*. Genes Dev..

[36] Yelina NE (2012). Epigenetic remodeling of meiotic crossover frequency in arabidopsis thaliana DNA methyltransferase mutants. PLoS Genet..

[37] Vincenten N (2015). The kinetochore prevents centromere-proximal crossover recombination during meiosis. eLife.

[38] Noh K-M (2015). Engineering of a histone-recognition domain in Dnmt3a alters the epigenetic landscape and phenotypic features of mouse ESCs. Mol. Cell.

[39] Dai J, Sullivan BA, Higgins JMG (2006). Regulation of mitotic chromosome cohesion by Haspin and aurora B. Dev. Cell.

[40] Dai J, Sultan S, Taylor SS, Higgins JM (2005). The kinase Haspin is required for mitotic histone H3 Thr 3 phosphorylation and normal metaphase chromosome alignment. Genes Dev..

[41] Wang S (2017). Inefficient crossover maturation underlies elevated aneuploidy in human female meiosis. Cell.

[42] Mather K (1939). Crossing over and heterochromatin in the X chromosome of Drosophila melanogaster. Genetics.

[43] Mahtani MM, Willard HF (1998). Physical and genetic mapping of the human X chromosome centromere: repression of recombination. Genome Res..

[44] Baumann C, Wang X, Yang L, Viveiros MM (2017). Error-prone meiotic division and subfertility in mice with oocyte-conditional knockdown of pericentrin. J. Cell Sci..

[45] Baumann C, Olson M, Wang K, Fazleabas A, De La Fuente R (2015). Arginine methyltransferases mediate an epigenetic ovarian response to endometriosis. Reproduction.

[46] Lehnertz B (2003). Suv39h-mediated histone H3 lysine 9 methylation directs DNA methylation to major satellite repeats at pericentric heterochromatin. Curr. Biol..

