# Supplementary material for: Helicase LSH/Hells regulates kinetochore function, histone H3/Thr3 phosphorylation and centromere transcription during oocyte meiosis
Source: Nat Commun. 2020 Sep 8;11:4486. doi: 10.1038/s41467-020-18009-3 (PMC7478982; doi:10.1038/s41467-020-18009-3)
Supplement: Supplementary file 1 — Supplementary Information [file 41467_2020_18009_MOESM1_ESM.pdf]

Nature Communications:

"Helicase LSH/Hells regulates kinetochore function, histone H3/Thr3 phosphorylation and centromere transcription during oocyte meiosis"

Baumann et al. 2020

Supplemetary Figures

Supplementary Figure 1

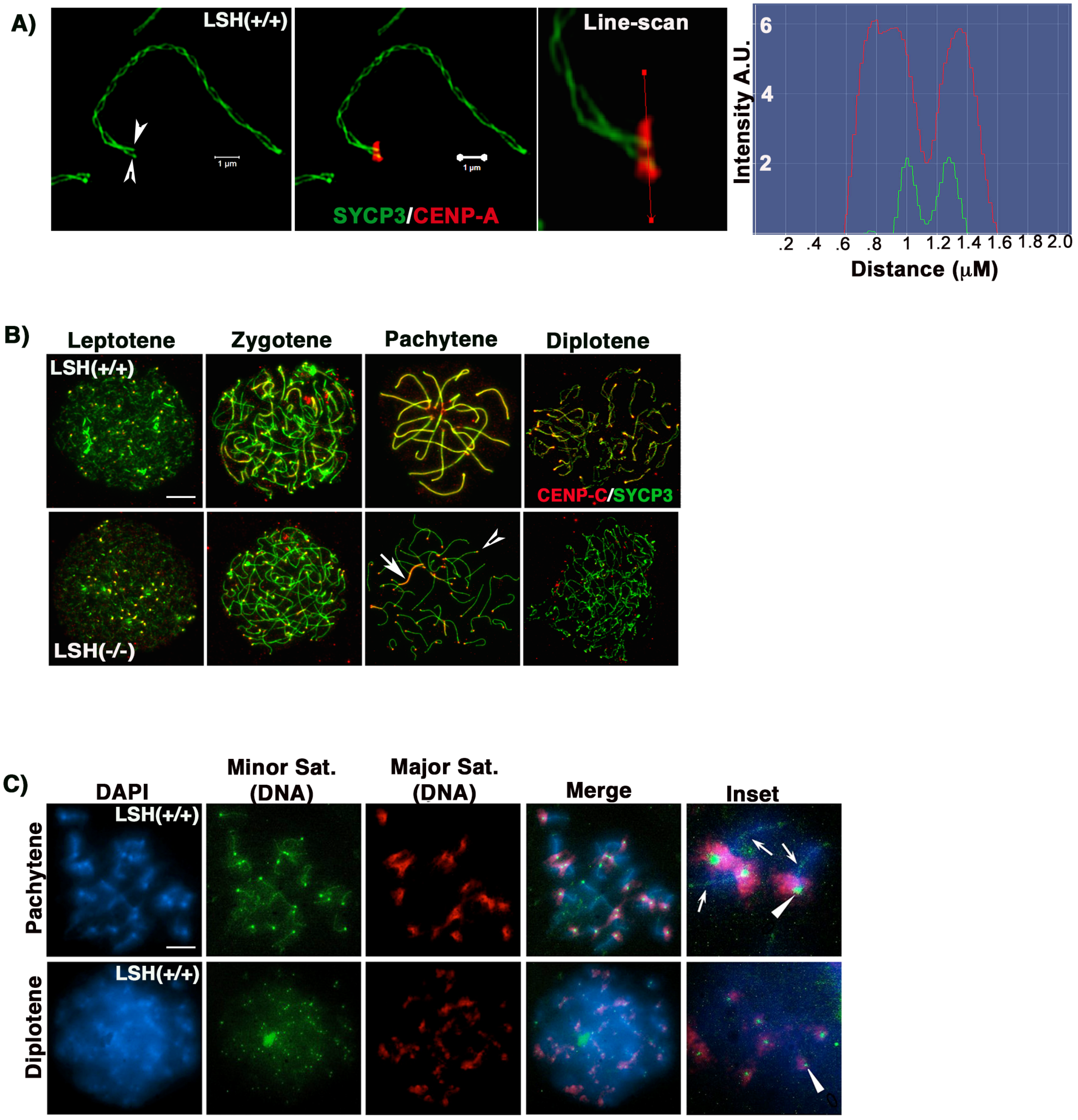

Supplementary Figure 1. Chromosomal localization of kinetochore proteins and minor satellite DNA sequences during oocyte meiotic prophase-I

A) Superresolution structured illumination (SR-SIM) resolves both strands (arrowheads) of the lateral elements o f the synaptonemal complex (green) in synapsed bivalents of wild-type oocytes at the pachytene stage. CENP-A (red) is specifically localized to the kinetochore domain of each strand. The line-scan reveals that the CENP-A signals (red peaks) extend beyond the SYCP3 signal (green peaks) resulting in kinetochore fusion. B) Dynamic chromosomal localization of CENP-C during meiotic prophase-I. In wild type oocytes, CENP-C is detected at the kinetochores at all stages of meiosis. However, it undergoes a transient localization to the synaptonemal complex at the pachytene stage. CENP-C is present at the kinetochores of LSH mutant oocytes but it fails to associate with the synaptonemal complex in Lsh-/- oocytes. Scale bar (5 µm). C) Analysis of major and minor satellite DNA sequences following fast hypotonic chromatin decondensation revealed that minor satellite sequences are detectable by fluorescence in situ hybridization (FISH) at the synaptonemal complex (arrows) in pachytene oocytes. Scale bar (5 µm).

Supplementary Figure 2

A)

		Mean	StDev	*P*-value
A01	Kdm1	0.36	1.42	4.77E-01
A02	Ash1l	0.53	1.41	5.96E-01
A03	Atf2	0.68	1.66	7.53E-01
A04	Aurka	-1.11	3.13	3.08E-01
A05	Aurkb	-1.26	2.47	1.89E-01
A06	Aurkc	2.21	0.89	7.86E-02
A07	Carm1	-0.31	1.60	2.29E-01
A08	Cdyl	-1.56	0.34	1.94E-04
A09	Ciita	0.55	1.55	6.39E-01
A10	Csrp2bp	0.39	1.50	5.22E-01
A11	Dnmt1	-0.41	2.46	3.75E-01
A12	Dnmt3a	0.64	1.44	6.88E-01
B01	Dnmt3b	0.36	1.61	5.28E-01
B02	Dot1l	-0.05	1.99	4.11E-01
B03	Dzip3	0.41	1.56	5.51E-01
B04	Ehmt1	0.44	1.45	5.40E-01
B05	Ehmt2	0.28	1.40	4.20E-01
B06	Esco1	-0.28	1.90	3.10E-01
B07	Esco2	0.34	2.76	6.99E-01
B08	Hat1	-0.24	2.12	3.71E-01
B09	Hdac1	-0.21	1.77	3.01E-01
B10	Hdac10	1.37	0.40	1.86E-01
B11	Hdac11	0.39	1.26	4.49E-01
B12	Hdac2	-1.42	0.44	7.02E-04
C01	Hdac3	0.69	1.69	7.63E-01
C02	Hdac4	0.47	1.44	5.62E-01
C03	Hdac5	0.36	1.26	4.28E-01
C04	Hdac6	0.60	1.71	7.10E-01
C05	Hdac7	0.63	1.44	6.84E-01
C06	Hdac8	0.43	1.33	4.98E-01
C07	Hdac9	-0.41	1.38	1.50E-01
C08	Kdm5b	0.46	1.49	5.64E-01
C09	Kdm5c	0.49	1.41	5.62E-01
C10	Kdm4a	0.49	1.52	5.92E-01
C11	Kdm4c	0.31	1.39	4.36E-01
C12	Kdm6b	1.35	0.36	1.69E-01
D01	Kat2a	0.34	1.37	4.54E-01
D02	Kat2b	-0.18	1.96	3.57E-01
D03	Kat5	-0.22	1.52	2.37E-01
D04	Mll3	-0.47	1.84	2.39E-01
D05	Mll5	0.41	1.31	4.82E-01
D06	Mysm1	0.73	1.66	7.92E-01
D07	Myst1	-0.40	1.36	1.48E-01
D08	Myst2	1.93	0.47	2.58E-02
D09	Myst3	0.34	1.38	4.54E-01
D10	Myst4	1.63	0.32	2.66E-02
D11	Ncoa1	-0.29	1.37	1.79E-01
D12	Ncoa3	0.83	1.78	8.80E-01
E01	Ncoa6	0.31	1.32	4.14E-01
E02	Nek6	0.96	1.73	9.69E-01
E03	Nsd1	0.83	1.82	8.82E-01
E04	Pak1	0.93	1.77	9.49E-01
E05	Prmt1	-1.20	0.04	7.32E-08
E06	Prmt2	1.38*	0.34	1.29E-01
E07	Prmt3	0.31	1.47	4.61E-01
E08	Prmt5	0.34	1.32	4.37E-01
E09	Prmt6	1.30	0.22	8.11E-02
E10	Prmt7	-0.33	1.97	3.08E-01
E11	Prmt8	-2.59	1.75	2.37E-02
E12	Rnf2	-0.53	1.40	1.33E-01
F01	Rnf20	0.68	1.61	7.46E-01
F02	Rps6ka3	0.39	1.40	4.95E-01
F03	Rps6ka5	1.39	0.25	5.56E-02
F04	Setd1a	1.65	0.76	2.15E-01
F05	Setd1b	-0.45	1.54	1.79E-01
F06	Setd2	0.61	1.51	6.81E-01
F07	Setd3	-0.26	1.36	1.83E-01
F08	Setd4	0.64	1.70	7.36E-01
F09	Setd5	0.41	1.46	5.24E-01
F10	Setd6	0.59	1.49	6.56E-01
F11	Setd7	0.40	1.32	4.77E-01
F12	Setd8	-0.74	1.62	1.37E-01
G01	Setdb1	0.67	1.55	7.33E-01
G02	Setdb2	2.13	1.76	3.26E-01
G03	Smyd1	0.71	1.78	7.94E-01
G04	Smyd3	-0.54	2.90	4.10E-01
G05	Suv39h1	-0.30	1.71	2.57E-01
G06	Suv420h1	-0.18	1.56	2.62E-01
G07	Ube2a	0.41	1.59	5.57E-01
G08	Ube2b	0.46	1.34	5.26E-01
G09	Usp16	0.25	1.49	4.32E-01
G10	Usp21	-1.48	4.90	4.31E-01
G11	Usp22	0.50	1.43	5.81E-01
G12	Whsc1	-0.30	1.71	2.59E-01

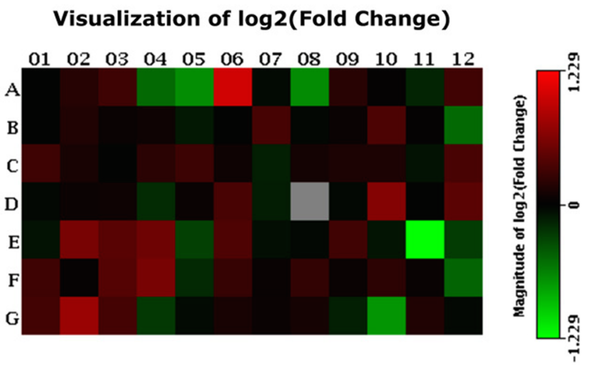

B)

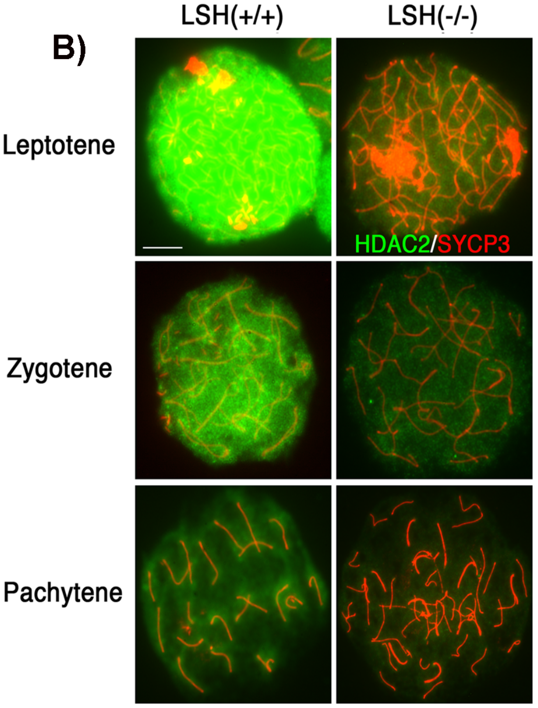

Supplementary Figure 2. Loss of LSH function affects the expression of chromatin modifying enzymes in prophase-I oocytes

A) Transcriptional profile of chromatin modifying enzymes in LSH mutant ovaries. Transcripts showing significant differences compared to wild-type gonads are highlighted (yellow). Data from 3 independent experimental replicates with representative heatmap. Unpaired t-Test, two-tailed resulted in the P-values indicated in the table. B) LSH (-/-) oocytes exhibit a significant reduction in the levels of histone deacetylase 2 (HDAC2) throughout meiotic prophase-I. Scale bar (10 μm).

Supplementary Figure 3

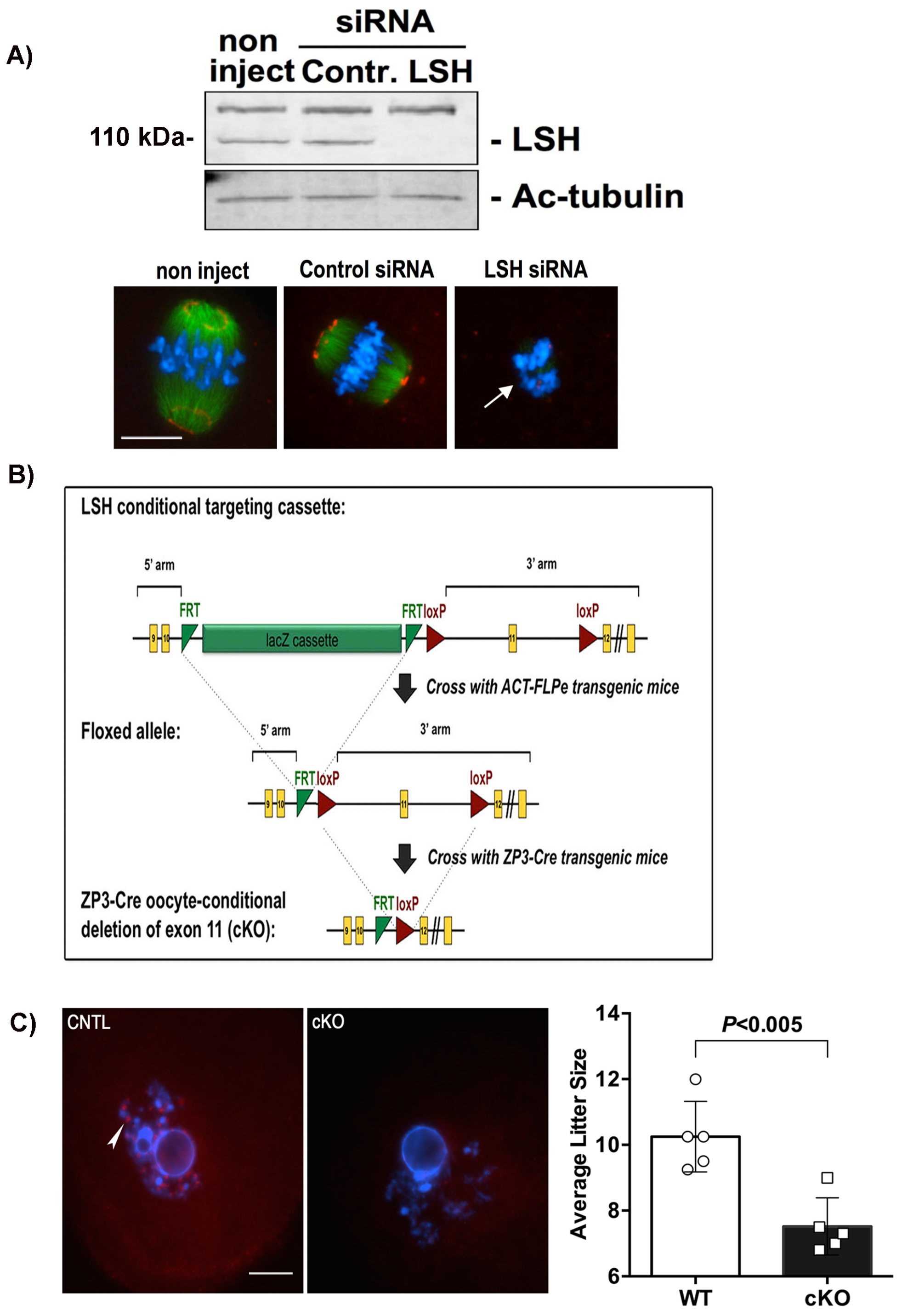

Supplementary Figure 3. Conditional deletion of LSH in pre-ovulatory oocytes induces abnormal chromosome segregation and subfertility

A) Analysis of LSH protein by western blot detects a 160 kDa band of unknown origin and a 110 kDa band that is specifically ablated by microinjection of a small inhibitory (siRNA) against Lsh transcripts. In the loading controls, no changes in the levels of the housekeeping protein, acetylated tubulin, were observed. Microinjection of a scrambled siRNA had no effect on the expression of LSH protein or chromosome alignment to the meiotic spindle. In contrast LSH siRNA induced severe chromosome segregation defects. Scale bar (25 µm). B) ZP3-Cre oocyte conditional deletion of LSH excised exon 11 disrupting the C-terminal helicase domain. Deletion of Exon 11-induced a frame shift mutation and introduces a premature STOP codon. C) Loss of LSH function in pre-ovulatory oocytes affects female fertility. LSH is present at nuclear speckles in wild-type GV stage oocytes. However no LSH protein is detected in conditional knockout (cKO) oocytes. Scale bar (10 µm). Average litter size in wild type and LSH cKO females after continuous mating to males of proven fertility. Data are expressed as the mean±s.d. litter size with (n=5) wild-type and (n=5) cKO females over a 6-month period. Statistical analysis was performed using unpaired t-Tests, two-tailed with P=0.0022.

Supplementary Figure 4

LSH cKO Oocytes

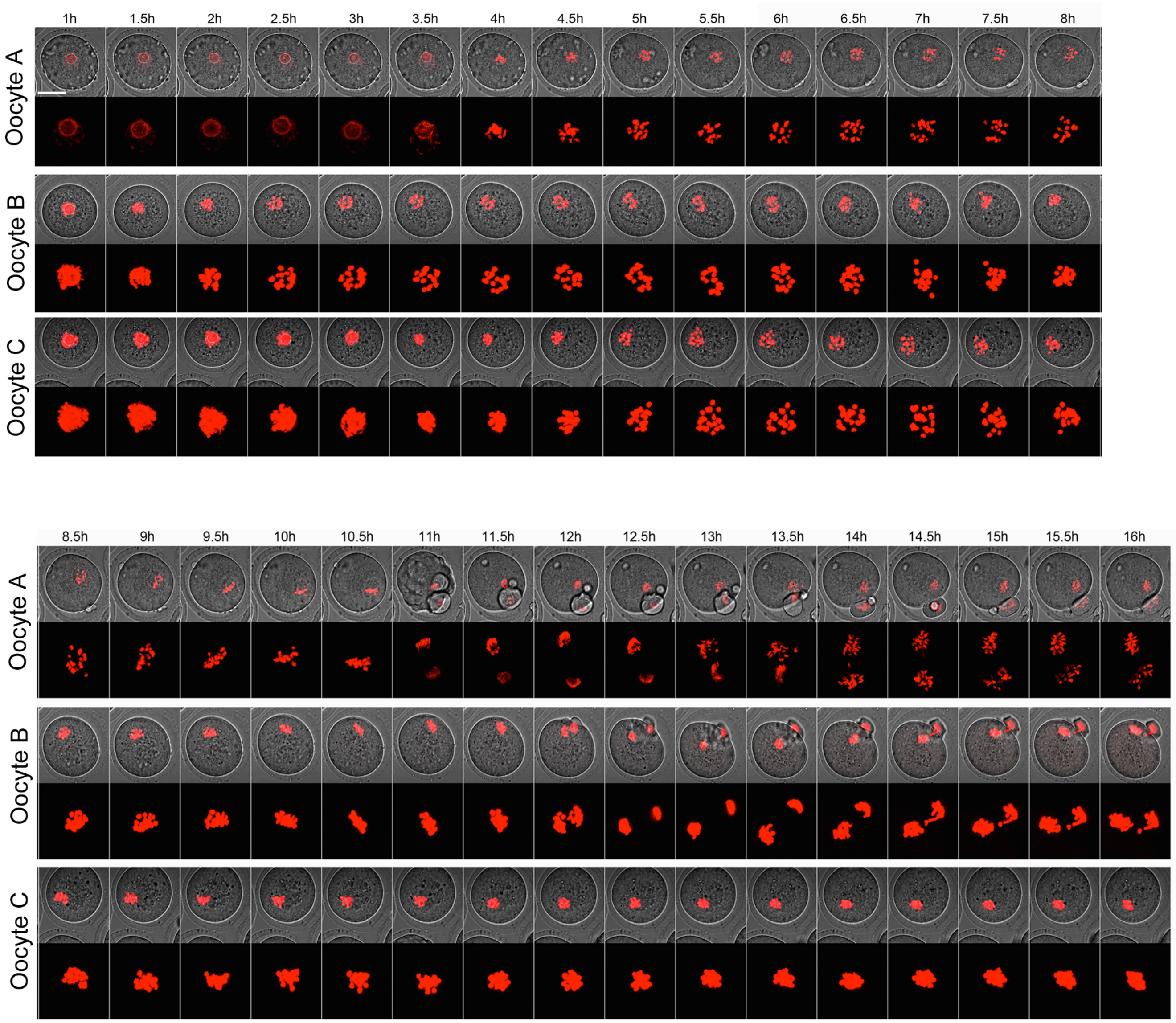

Supplementary Figure 4. Time-lapse analysis of oocyte maturation in LSH cKO oocytes. Mutant pre-ovulatory oocytes were microinjected with RFP-H2B and allowed to undergo in vitro maturation for 16 hours. Individual DIC and confocal images are presented at indicated times for three representative oocytes. Scale bar (40 μm).

Supplementary Figure 5

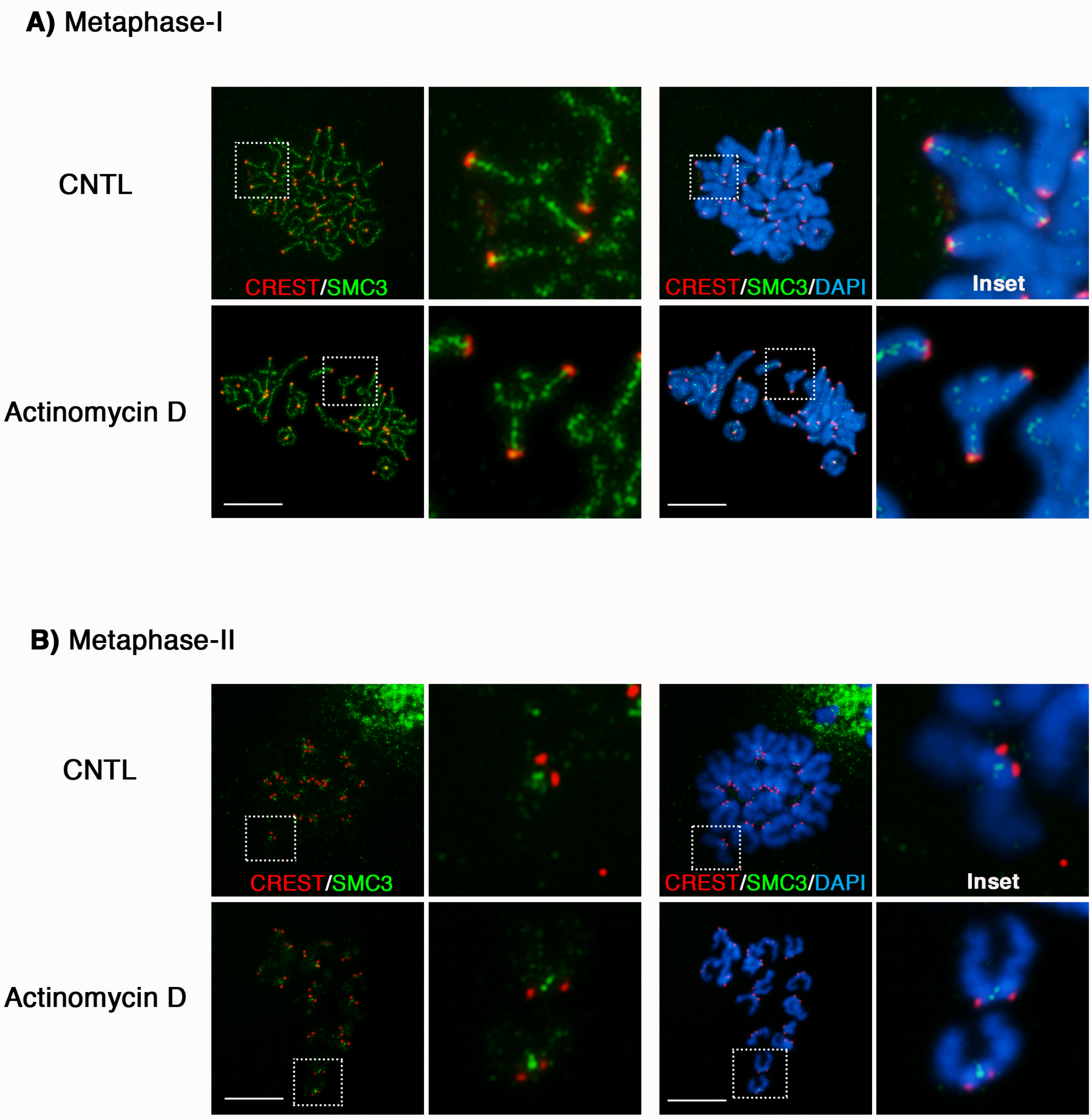

Supplementary Figure 5. The condensin protein SMC3 remains localized to the inter chromatid space in metaphase-I and inner centromere of metaphase-II chromosomes following transcriptional inhibition. A) Treatment of metaphase-I oocytes with the transcriptional inhibitor Actinomycin D (1 µg/ml/1h) had no effect on the localization of SMC3 to the inter chromatid space in metaphase-I bivalents. B) SMC3 remains localized at the inner centromere following treatment with Actinomycin D at the metaphase-II stage. Scale bars (5 µm).

Supplementary Figure 6

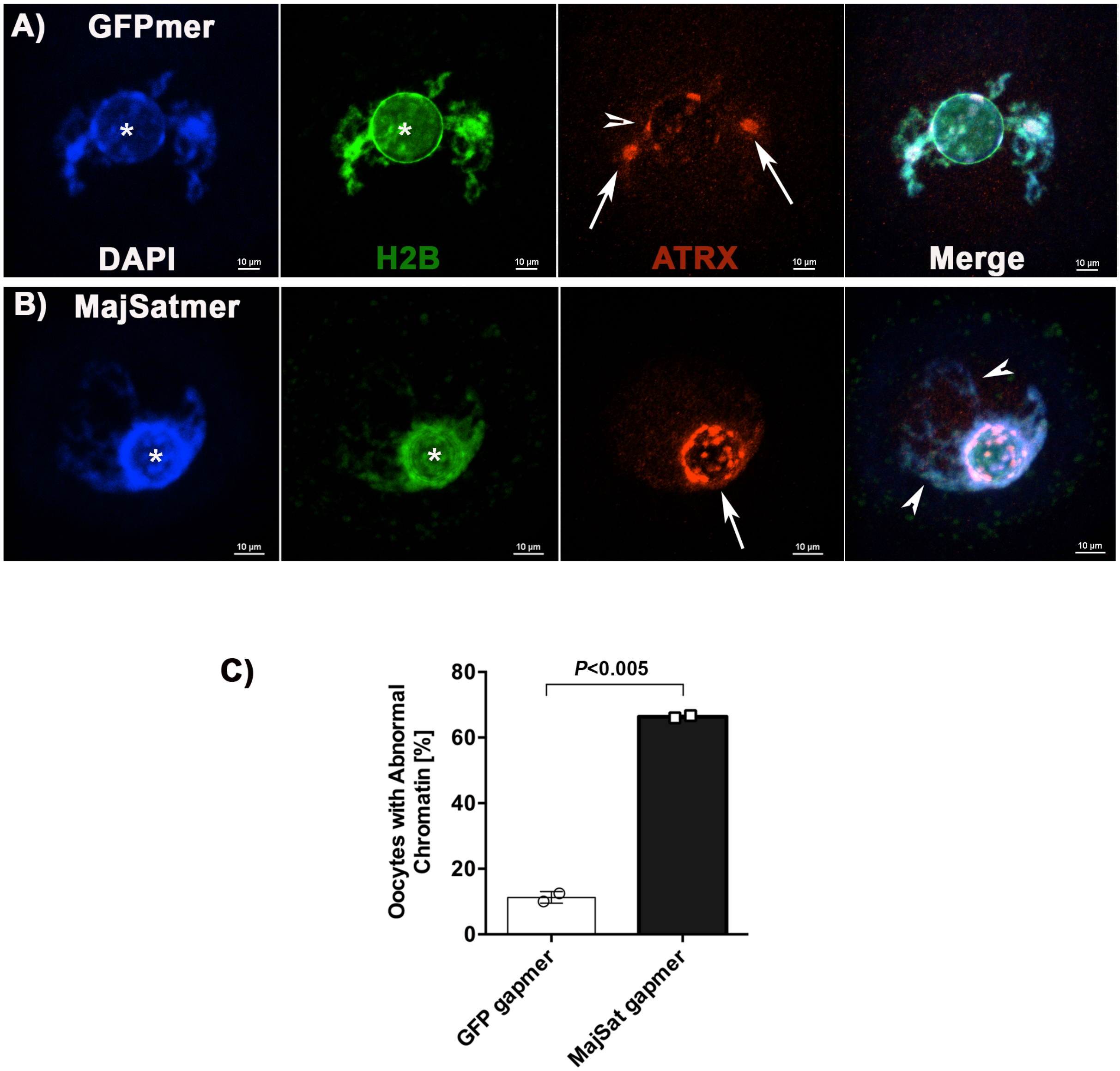

Supplementary Figure 6. Knockdown of major satellite transcripts induced abnormal chromatin configuration in GV stage oocytes. A) Control germinal vesicle (GV) stage oocyte microinjected with locked nucleic acid LNA-DNA GFP gapmer. Chromatin is stained with an anti-histone H2B nanobody (green). Chromocenters are stained with an anti-ATRX antibody (red). Chromatin exhibits the typical surrounded nucleolus configuration with small chromocenters in close apposition with the nucleolus (arrowhead) and large chromocenters associated with distant condensed chromatin fibers (arrows). The position of the nucleolus is indicated by (*). B) Microinjection of LNA-DNA MajSat gapmers to knockdown forward and reverse satellite transcripts induced abnormal chromatin configuration in which chromocenters become associated with the nucleolus (arrow) with formation of thin chromatin fibers that stretch to different extent into the germinal vesicle (arrowheads).
C) Proportion of GV stage oocytes that exhibited abnormal chromatin configuration after 44 h of culture at the GV stage in control GFP gapmer (n=18) and MajSat Fwd/Rev gapmer microinjected (n=24) groups. Data from two independent experimental replicates. *** P=0.0006. Scale bars=10 μM.
