# Supplementary material for: Helicase LSH/Hells regulates kinetochore function, histone H3/Thr3 phosphorylation and centromere transcription during oocyte meiosis
Source: Nat Commun. 2020 Sep 8;11:4486. doi: 10.1038/s41467-020-18009-3 (PMC7478982; doi:10.1038/s41467-020-18009-3)
Supplement: Supplementary file 2 — Description of Additional Supplementary Files [file 41467_2020_18009_MOESM2_ESM.pdf]

Description of Additional Supplementary Files

Supplementary Data 1: PCR Array. Fold change values for all epigenetic chromatin modifiers detected in LSH-$^{/-}$ ovaries compared to wildtype controls (three replicates) with mean, s.d. and P<values indicated.

Supplementary Data 2: Key reagents. A list of key reagents and their sources.

Supplementary Movies 1-2: 3D-Superresolution kinetochore analysis of wild-type (WT) and LSH knockout (KO) oocytes at meiotic prophase-I. Meiotic kinetochores were stained with CENP-A and analyzed with superresolution structured illumination (SR-SIM)**.** 3-D renderings from the kinetochores of wild-type and LSH KO pachytene oocytes were generated by analysis of 85 individual Z-scans (0.091 µM) each and reconstructed using NIS Elements 4.0. The imaging software assigns a different color to each pair of fused kinetochores. Wild-type oocytes exhibit fused kinetochores. LSH KO oocytes exhibit significantly decondensed fused kinetochores in addition to several kinetochores that fail to fuse at the pachytene stage.

Supplementary Movie 3: LSH localization to the meiotic spindle poles in Metaphase-II stage oocytes. LSH (red) is co-localized with Pericentrin (green) at the meiotic spindle poles in metaphase-II oocytes. 3-D rendering of a single spindle pole from a wild-type oocyte generated after analysis of 13 individual Z-scans (0.091 µM) each and reconstructed using SR-SIM (Zen) software.

Supplementary Movies 4-5: Live cell imaging of oocyte maturation and chromosome segregation in WT and LSH conditional knockout (cKO) oocytes. Wild-type pre-ovulatory oocytes at the GV stage were microinjected with capped mRNA encoding for GFP-H2B and

matured in vitro for 16 h. Control oocytes exhibit extrusion of the first polar body and timely reach the metaphase-II stage with no evidence for chromosome segregation defects. LSH cKO oocytes at the GV stage were microinjected with capped mRNA encoding for RFP-H2B. LSH mutant oocytes frequently exhibit a metaphase-I arrest even after 20 h of in vitro maturation.

Supplementary Movies 6-7: Super resolved 3-D structured illumination reveals abnormal kinetochore structure in Lsh$^{-/-}$ oocytes at metaphase-I. Meiotic kinetochores from metaphase-I oocytes were stained with CREST and analyzed with SR-SIM. 3-D renderings from the kinetochores of wild-type and LSH cKO oocytes at the metaphase-I were generated by analysis of 22 (WT) and 71 (LSH cKO) individual Z-scans (0.091 µM interval) each and reconstructed using NIS Elements 4.0. Wild-type oocytes exhibit fused and highly compact kinetochores. LSH cKO oocytes exhibit significantly decondensed kinetochores and abnormal kinetochore structure at the metaphase-I stage.
