# Supplementary material for: Helicase LSH/Hells regulates kinetochore function, histone H3/Thr3 phosphorylation and centromere transcription during oocyte meiosis
Source: Nat Commun. 2020 Sep 8;11:4486. doi: 10.1038/s41467-020-18009-3 (PMC7478982; doi:10.1038/s41467-020-18009-3)
Supplement: Supplementary file 4 — Supplementary Data 2 [file 41467_2020_18009_MOESM4_ESM.pdf]

Supplementary Data 2

Mouse strains	Source	ID#
LSH knockout first mice C57BL/6NTac-Hellstm1a(EUCOMM)Wtsi/leg	EUCOMM	EPD0102_4_A04
Act-FLPe mice (B6;SJL-Tg(ACTFLPe)9205Dym/J)	The Jackson Lab	005703

Antibodies	Supplier	Catalog #
LSH (Smarca-6) - rabbit polyclonal (1:500)	abcam, Cambridge, MA	ab3851
anti-CREST antiserum (ANA-Centromere Autoantibody) - (1:500)	Cortex Biochem, Concord, MA	CS1058
CENP-A - rabbit polyclonal (1:500)	Cell Signaling Technologies, Danvers, MA	CST2186
SYCP3 - mouse monoclonal (1:500)	abcam, Cambridge, MA	ab97672, COR 10G11/7
CENP-C - rabbit polyclonal (1:200)	a generous gift from Bill Earnshaw	-
anti-phospho-histone H3T3 (H3T3ph) rabbit polyclonal (1:400)	EMD Millipore	07-424
5mC, mouse monoclonal (1:200)	Calbiochem	NA81
Dnmt1 (pATH52), 1:200)	a generous gift from Dr. Tim Bestor	-
HDAC2, rabbit polyclonal (1:200)	abcam, Cambridge, MA	ab16032
SMC3, rabbit polyclonal (1:200)	abcam, Cambridge, MA	ab9263
acetylated alpha-tubulin, mouse monoclonal (1:1000)	Sigma, St. Louis, MO	T6793, clone 6-11B-1
RNA Polymerase II phospho S2 (RNA-PolII-S2) - rabbit polyclonal (1:200)	abcam, Cambridge, MA	ab5095
beta-tubulin, mouse monoclonal (1:2000)	Sigma, St. Louis, MO	T4026, clone TUB 2.1
Pericentrin (Pcnt) - mouse polyclonal (1:400)	BD Biosciences, San Diego, CA	611814, clone 30
rabbit anti-LSH antibody (1:5000)	a generous gift from Kathrin Muegge	-
Peroxidase-conjugated goat-anti-rabbit (1:5,000)	Jackson Immuno Research, PA	111-036-144
Peroxidase-conjugated goat-anti-mouse (1:50,000)	Jackson Immuno Research, PA	115-036-062
alpaca/recombinant VhH domain nanobody against histone H2B ATTO488-conjugated (Histone-Label) (1:200)	ChromoTek, Germany	tba488-100
Anti-rabbit polyclonal against ATRX (H-300) (1:200)	Santa Cruz, TX	sc-15408

Inhibitors	Supplier	Catalog #
Alpha-amanitin (50µg/ml for 17h)	Sigma, St. Louis, MO	A2263
Actinomycin D (1µg/ml for 1h)	Sigma, St. Louis, MO	A9415

FISH probes	Supplier	Catalog #
StarFISH Concentrated Mouse Pan-Centromeric Probe, CY3 labeled	Cambio Ltd., Cambridge, UK	1697-MCY3-02

Oligos/Assays/Arrays	Reference/Supplier (Cat#)
RT² qPCR Primer Assay for Mouse Hells	Qiagen (PPM57658A)
RT² qPCR Primer Assay for Mouse Gapdh	Qiagen (PPM02946E)
RT² Profiler™ PCR Array Mouse Epigenetic Chromatin Modification Enzymes	Qiagen (PAMM-085Z)
MajSat-Fwd: 5'-*GACGACTTGAAAAATGACGAAATC*-3'<br>MajSat-Rev: 5'-*CATATTCCAGGTCCTTCAGTGTGC*-3'	Lehnertz et al. Curr. Biol. 2003
Beta-actin-Fwd: 5'-*GGCACCACACCTTCTACAATG*-3'<br>Beta-actin-Rev: 5'-*GTGGTGGTGAAGCTGTAGCC*-3'	Housekeeping Control
LSH-flip-Fwd: 5'-*CGGAAAACAGTATCTTCAGGATGGA*-3'<br>LSH-flip-Rev: 5'-*CAGGCCAGCTTGACTCAAAT*-3'	Wild type allele/loxP recombination verification
CRE-Fwd: 5'-*CATTTGGGCCAGCTAAACAT*-3'<br>CRE-Rev: 5'-*TAAGCAATCCCCAGAAATGC*-3'	EUCOMM for presence of CRE
tm1d-Fwd: 5'-*AAGGCGCATAACGATACCAC*-3'<br>tm1d-Rev: 5'-*ACTGATGGCGAGCTCAGACC*-3'	EUCOMM for tm1d conversion
loxP-Fwd: 5'-*ATCCGGGGGTACCGCGTCGAG*-3'<br>loxP-Rev: 5'-*ACTGATGGCGAGCTCAGACC*-3'	EUCOMM for presence of loxP
LossE11-Fwd: 5'-*AAGTGCCGGCTAATCAGGGA*-3'<br>LossE11-Rev: 5'-*AGGACATGTATGCTGCCTGG*-3'	EUCOMM for loss of Exon 11

LNA-DNA Gapmers	Source	Sequence
LNA DNA gapmer maj Fwd	Qiagen	acatCCACTTGACGActtg
LNA DNA gapmer maj Rev	Qiagen	tattTCACGTCCTAAagtg
LNA DNA gapmer GFP	Qiagen	gagaAAGTGTGACAagtg

Vectors	Source	Catalog #
pGEMHE-H2B-mRFP	Euroscarf (deposited by Dr. J. Ellenberg)	P30517
pIVT-H2B-GFP	a generous gift from Dr. Richard Schultz	
