# Supplementary material for: Helicase LSH/Hells regulates kinetochore function, histone H3/Thr3 phosphorylation and centromere transcription during oocyte meiosis
Source: Nat Commun. 2020 Sep 8;11:4486. doi: 10.1038/s41467-020-18009-3 (PMC7478982; doi:10.1038/s41467-020-18009-3)
Supplement: Supplementary file 12 — Reporting Summary [file 41467_2020_18009_MOESM12_ESM.pdf]

nature research

Corresponding author(s): Rabindranath De La Fuente

Last updated by author(s): Jun 22, 2020

Reporting Summary

Nature Research wishes to improve the reproducibility of the work that we publish. This form provides structure for consistency and transparency in reporting. For further information on Nature Research policies, see Authors & Referees and the Editorial Policy Checklist .

Statistics

For all statistical analyses, confirm that the following items are present in the figure legend, table legend, main text, or Methods section.

n/a	Confirmed	
☐	☒	The exact sample size (n) for each experimental group/condition, given as a discrete number and unit of measurement
☐	☒	A statement on whether measurements were taken from distinct samples or whether the same sample was measured repeatedly
☐	☒	The statistical test(s) used AND whether they are one- or two-sided<br>*Only common tests should be described solely by name; describe more complex techniques in the Methods section.*
☒	☐	A description of all covariates tested
☐	☒	A description of any assumptions or corrections, such as tests of normality and adjustment for multiple comparisons
☐	☒	A full description of the statistical parameters including central tendency (e.g. means) or other basic estimates (e.g. regression coefficient) AND variation (e.g. standard deviation) or associated estimates of uncertainty (e.g. confidence intervals)
☐	☒	For null hypothesis testing, the test statistic (e.g. F, t, r) with confidence intervals, effect sizes, degrees of freedom and P value noted<br>*Give P values as exact values whenever suitable.*
☒	☐	For Bayesian analysis, information on the choice of priors and Markov chain Monte Carlo settings
☒	☐	For hierarchical and complex designs, identification of the appropriate level for tests and full reporting of outcomes
☒	☐	Estimates of effect sizes (e.g. Cohen's d, Pearson's r), indicating how they were calculated

Our web collection on statistics for biologists contains articles on many of the points above.

Software and code

Policy information about availability of computer code

Data collection	Data in this manuscript are predominantly microscopy data. These data were collected using commercial software that are associated with the microscopes used: Improvision Openlab 3.1.7; Nikon ECZ1 3.91 and Zeiss ZEN 2.3
Data analysis	For data analysis, we used Graphpad Prism 6., which is available at https://www.graphpad.com, as well as MS Excel 14.4.8 and Nikon NIS Elements 4.0

For manuscripts utilizing custom algorithms or software that are central to the research but not yet described in published literature, software must be made available to editors/reviewers. We strongly encourage code deposition in a community repository (e.g. GitHub). See the Nature Research guidelines for submitting code & software for further information.

Data

Policy information about availability of data

All manuscripts must include a data availability statement. This statement should provide the following information, where applicable:

- Accession codes, unique identifiers, or web links for publicly available datasets
- A list of figures that have associated raw data
- A description of any restrictions on data availability

The data that support the findings of this study are available from the corresponding author upon reasonable request.

Field-specific reporting

Please select the one below that is the best fit for your research. If you are not sure, read the appropriate sections before making your selection.

Life sciences study design

All studies must disclose on these points even when the disclosure is negative.

Sample size	The number of samples/experimental replicates for each data panel has been described in the manuscript. We chose sample sizes based on previous studies from our and other laboratories indicating that our sample sizes per replicate have sufficient power to distinguish statistical significance after three replicates.
Data exclusions	No data were excluded from the analyses.
Replication	All experimental findings were two to five times successfully replicated. Details are outlined in the Methods section of the manuscript and each figure legend.
Randomization	Randomization is not relevant to this study, as we compared genetically modified or chemically treated samples with wild type controls, respectively.
Blinding	Most experimental procedures and quantification of results were done by at least two independent researchers. The observed phenotype in LSH knockout oocytes is so severe (>90% oocytes show defects) that the genotype is immediately obvious and blinding was, thus, discontinued after the initial observations.

Reporting for specific materials, systems and methods

We require information from authors about some types of materials, experimental systems and methods used in many studies. Here, indicate whether each material, system or method listed is relevant to your study. If you are not sure if a list item applies to your research, read the appropriate section before selecting a response.

Materials & experimental systems

n/a	Involved in the study	
☐	☒	Antibodies
☒	☐	Eukaryotic cell lines
☒	☐	Palaeontology
☐	☒	Animals and other organisms
☒	☐	Human research participants
☒	☐	Clinical data

Methods

n/a	Involved in the study	
☒	☐	ChIP-seq
☒	☐	Flow cytometry
☒	☐	MRI-based neuroimaging

Antibodies

Antibodies used	All antibodies used in the manuscript have been fully described in the materials and methods and supplemental table 1 and are listed here: ab3851 (LSH/Smarca-6) abcam; CS1058 (CREST) Cortex Biochem; CST2186 (CENP-A) Cell Signaling Technologies; ab97672 (SYCP3) abcam; CENP-C a gift from Bill Earnshaw; 07-424 (H3T3ph) EMD Millipore; NA81 (5mC) Calbiochem; DNMT1 a gift from Tim Bestor; ab16032 (HDAC2) abcam; ab9263 (SMC3) abcam; T6793 (acetylated alpha-tubulin) Sigma; ab5095 (RNA PolII) abcam; T4026 (beta-tubulin) Sigma; 611814 (Pericentrin) BD Biosciences; LSH a gift from Kathrin Muegge; 111-036-144 (peroxidase-conjugated goat-anti-rabbit) Jackson Immuno Research; 115-036-062 (peroxidase-conjugated goat-anti-rabbit) Jackson Immuno Research; tba488-100 (H2B Atto nanobody) ChromoTek; sc-15408 (ATRX) Santa Cruz;
Validation	All antibodies are validated. Information is provided on the manufacturers' websites and here:<br>ab3851: abcam Abpromise guarantee covers the use of ab3851 in the following tested applications: ICC/IF, WB 1/2000 - 1/10000. Detects a band of approximately 97 kDa (predicted molecular weight: 97 kDa) and IP.<br>CS1058: tested applications: ICC/IF<br>CST2186: CENP-A Antibody detects endogenous CENP-A protein. This antibody does not cross-react with other histone proteins, including Histone H3. tested application: WB, ICC/IF<br>ab97672: abcam Abpromise guarantee covers the use of ab97672 in the following tested applications: IHC-P, ELISA, ICC/IF, IHC-Fr, WB<br>07-424: EMD Millipore Performance Guarantee for tested applications: IF, WB, ICC<br>NA81: Detects methylated DNA from a broad range of species. Tested Applications: Flow Cytometry, Frozen Sections, Immunoblotting, Immunofluorescence, Paraffin Sections<br>ab16032: abcam Abpromise guarantee covers the use of ab16032 in the following tested applications: IHC-Fr, IHC-P, IHC-Fr, ICC/IF, WB, IP<br>ab9263: abcam Abpromise guarantee covers the use of ab16032 in the following tested applications: IP, WB, IHC-P, ICC/IF<br>T6793: tested for dot blot, electron microscopy, immunocytochemistry, indirect ELISA, radioimmunoassay, western blot<br>ab5095: abcam Abpromise guarantee covers the use of ab16032 in the following tested applications: ELISA, IHC-FoFr, ChIP, ChIP/Chip, IHC-P, IHC-Fr, ICC/IF, IHC - Wholemount, Dot blot, WB, CHIPseq<br>T4026: tested for indirect immunofluorescence, western blot<br>611814: tested for use in Western blot, Immunofluorescence

111-036-144: Based on immunoelectrophoresis and/or ELISA, the antibody reacts with whole molecule rabbit IgG. It also reacts with the light chains of other rabbit immunoglobulins. No antibody was detected against non-immunoglobulin serum proteins. The antibody has been tested by ELISA and/or solid-phase adsorbed to ensure minimal cross-reaction with human, mouse and rat serum proteins, but it may cross-react with immunoglobulins from other species.

115-036-062: Based on immunoelectrophoresis and/or ELISA, the antibody reacts with whole molecule mouse IgG. It also reacts with the light chains of other mouse immunoglobulins. No antibody was detected against non-immunoglobulin serum proteins. The antibody has been tested by ELISA and/or solid-phase adsorbed to ensure minimal cross-reaction with human, bovine and horse serum proteins, but it may cross-react with immunoglobulins from other species.

tba488-100: consists of an anti-histone VHH conjugated to the fluorescent dye ATTO488 tested for immunofluorescence (IF/ICC) and Super Resolution Microscopy (SRM) microscopy of histones, chromosomes, and nuclei, conjugated with ATTO488, specific to Histone H2A-H2B heterodimers

sc-15408: tested for IF, WB

Animals and other organisms

Policy information about studies involving animals; ARRIVE guidelines recommended for reporting animal research

Laboratory animals	Male and female C57BL/6NTac-Hellstm1a(EUCOMM)Wtsi/leg; B6;SJL-Tg(ACTFLPe)9205Dym/J; C57BL/6-Tg(Zp3-cre)93Knw/J; mice were obtained from the European Conditional Mouse Mutagenesis Program or were purchased from The Jackson Laboratory. The genetically modified animals are on C57BL/6 background. Experiments were conducted on a) times mated embryos on dpc 18.5, or b) juvenile female mice on days 20-24 pp.
Wild animals	The study did not involve wild animals.
Field-collected samples	The study did not involve samples collected from the field.
Ethics oversight	This study was approved by and performed in accordance to guidelines of the University of Georgia Institutional Animal Care and Use Committee (IACUC).

Note that full information on the approval of the study protocol must also be provided in the manuscript.
