# Supplementary material for: Helicase LSH/Hells regulates kinetochore function, histone H3/Thr3 phosphorylation and centromere transcription during oocyte meiosis
Source: Nat Commun. 2020 Sep 8;11:4486. doi: 10.1038/s41467-020-18009-3 (PMC7478982; doi:10.1038/s41467-020-18009-3)

Source Data 1

A) Western blot Figure 4a

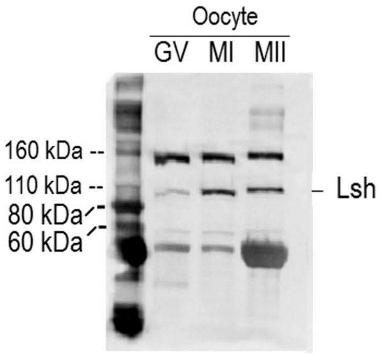

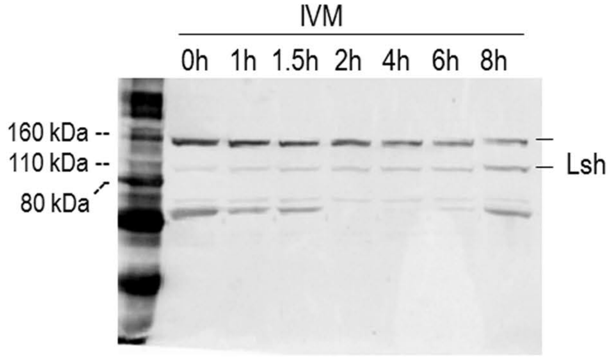

B) Western blot Figure 4c

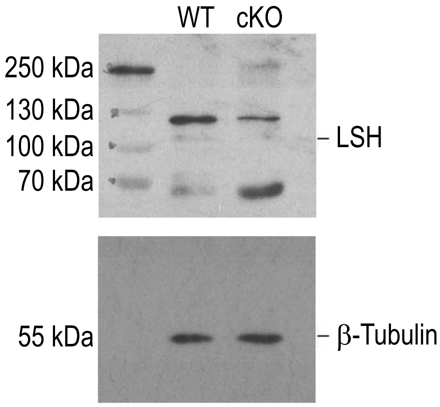

C) Western blot Figure 6a

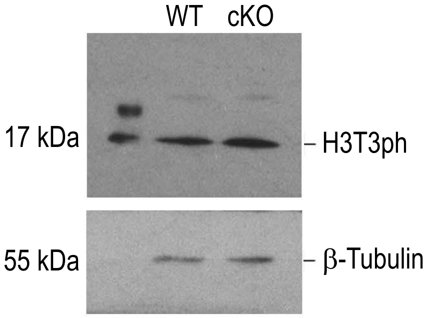

D) Western blot Supplemental Figure 3a

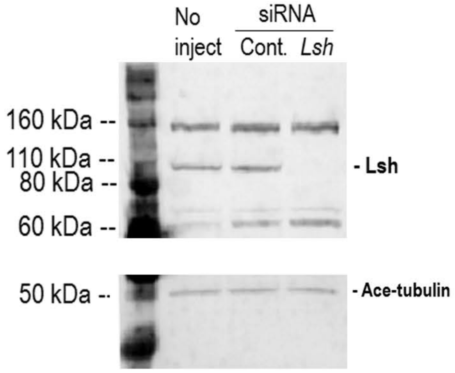

Supplement: Supplementary file 13 — Source Data [file 41467_2020_18009_MOESM13_ESM.pdf]
